# Heterodimer Binding Scaffolds Recognition via the Analysis of Kinetically Hot Residues

**DOI:** 10.3390/ph11010029

**Published:** 2018-03-16

**Authors:** Ognjen Perišić

**Affiliations:** 1 Big Blue Genomics, Vojvode Brane 32, 11000 Belgrade, Serbia; ognjen.perisic@gmail.com; 2 Department of Chemistry, New York University, 1001 Silver, 100 Washington Square East, New York, NY 10003, USA

**Keywords:** protein-protein interactions, normal mode analysis, Gaussian Network Model, protein decoys

## Abstract

Physical interactions between proteins are often difficult to decipher. The aim of this paper is to present an algorithm that is designed to recognize binding patches and supporting structural scaffolds of interacting heterodimer proteins using the Gaussian Network Model (GNM). The recognition is based on the (self) adjustable identification of kinetically hot residues and their connection to possible binding scaffolds. The kinetically hot residues are residues with the lowest entropy, i.e., the highest contribution to the weighted sum of the fastest modes per chain extracted via GNM. The algorithm adjusts the number of fast modes in the GNM’s weighted sum calculation using the ratio of predicted and expected numbers of target residues (contact and the neighboring first-layer residues). This approach produces very good results when applied to dimers with high protein sequence length ratios. The protocol’s ability to recognize near native decoys was compared to the ability of the residue-level statistical potential of Lu and Skolnick using the Sternberg and Vakser decoy dimers sets. The statistical potential produced better overall results, but in a number of cases its predicting ability was comparable, or even inferior, to the prediction ability of the adjustable GNM approach. The results presented in this paper suggest that in heterodimers at least one protein has interacting scaffold determined by the immovable, kinetically hot residues. In many cases, interacting proteins (especially if being of noticeably different sizes) either behave as a rigid lock and key or, presumably, exhibit the opposite dynamic behavior. While the binding surface of one protein is rigid and stable, its partner’s interacting scaffold is more flexible and adaptable.

## 1. Introduction

The revolutions in biotechnology of the past two decades opened an unprecedented ability to analyze and organize biological information. The advent of the next generation sequencing technologies and accompanying software tools enables the sequencing and analysis of complete genomes, not only of whole species but of individual specimens also, often at a single cell level [1,2,3]. More than 90 million protein sequences have been deciphered so far, and that number grows at an enormous rate [4], but the sequencing data alone is not sufficient to fully grasp the biological process on the molecular level. The detailed information on structural and physical interactions of biological molecules is of the utmost importance for the understanding of biological processes and their proper treatment. However, the capacity to generate and adequately connect structural data, i.e., protein, DNA, and RNA structures, to biological processes is diminutive in comparison to the sequencing yield or even to diagnostic abilities. The analysis of human proteome reveals that almost half of human genes and more than 60% of metabolic enzymes are expressed in majority of tissues [5], but for the majority of them roles and interactions are still unknown. An even more pressing issue, and one that is very related to structural and sequencing information, is the high attrition rate in drug development, a conclusion drawn in 2004 [6]. The past decade did not rectify this issue, as explained in [7]. New approaches, such as the one described in this manuscript, or the analysis of ligand binding behavior within a framework of chemico-biological space [8], may be a way toward a much better compound filtering during preclinical trials and thus toward a more efficient drug design.

To fully comprehend the biological process on the molecular level we first have to understand the physical laws that govern the interactions of biological polymers. Protein-DNA and protein-lipid interactions had been successfully addressed [9,10,11,12,13], but the problems of protein folding [14,15] and protein-protein interactions [16,17,18,19,20,21] are issues that still require the full attention of the research community. Many attempts were made to develop a comprehensive protein-protein interaction theory. The recognition of binding residues using an analysis of sequential and structural properties of heteromeric, transient protein-protein interactions produced very good overall results, as shown by Neuvirth et al. [22]. Chen and Zhou [23] used sequence profiles, as well as solvent accessibility of spatially neighboring surface residues fed to neural networks to develop a successful binding sites recognition protocol. By applying a linear combination of the energy score, interface propensity, and residue conservation score, Liang et al. [24] achieved decent coverage and accuracy. Zhang et al. focused their effort on the interface conservation across structure space [25], while Saccà et al. introduced multilevel (protein, domain, and residue) binding recognition using the Semantic Based Regularization Machine Learning framework [26]. It was shown that the three-dimensional structural information, either based on data from PDB [27] or obtained from homology modeling, produces a robust and efficient prediction of protein-protein interactions when applied with information on structural neighbors of queried proteins and Bayesian classifiers [28]. The protein (co)expression also attracts researchers’ attention. For instance, Bhardwaj and Lu [29] showed that the complexity of co-expression profiles in protein networks rises with the increase of the interactions/connectedness of the networks. 

The application of coarse-grained force fields in the analysis of protein-protein associations also attracted the attention of the research community [19,30]. Basdevant, Borgis, and Ha-Duong analyzed the dimer association using the coarse-grained SCORPION force field model of protein and solvent [31]. The force field model was able to recognize near native decoys of three different protein complexes (out of thousands of analyzed decoys) and to efficiently simulate the dynamics of recognition of a protein complex starting from different initial structures. A similar approach, in a combination with a push-pull-release sampling strategy, was applied by Ravikumar, Huang, and Yang to examine protein-protein association in a number of complexes [32]. M. Zacharias combined bonded atomistic with coarse-grained, non-bonded interactions in his force-field model to simulate peptide-protein docking and refinement from different stating geometries with acceptable accuracy [33]. Solernou and Fernandez-Recio developed pyDockCG [34], a coarse-grained potential for protein-protein docking scoring and refinement, based on the earlier UNRES model developed for the protein structure prediction [35]. A coarse-grained approach (one pseudo atom per every three residues) by Frembgen-Kesner and Elcock showed an ability to reproduce the absolute association rate constants of wild-type and mutant protein pairs via Brownian motion simulations when hydrodynamic interactions between diffusing proteins are included [36]. Chou and collaborators used Pseudo Amino Acid Composition (PseAAC) and Wavelet Transforms as inputs to predict algorithms based on Random forests, as well as Support Vector Machines to recognize protein binding sites [37,38,39,40,41,42,43].

The elucidation of physical interactions of proteins is appealing to the pharmaceutical industry as well [44], with an emphasis on small molecule inhibitors of protein-protein interactions [45,46]. The interest of the pharmaceutical industry is not surprising, because mutations, which disrupt the three-dimensional structure, can be cancer drivers [47]. 

The aim of this paper is to address the physical interactions between individual protein chains that form protein dimers. The approach described here uses the structural information only and the theory of phantom networks through its Gaussian network model (GNM) implementation [48,49,50,51,52,53,54,55,56,57,58,59]. The GNM produces a set of vibrational modes via the eigenvalues and eigenvectors of the protein Kirchhoff contact matrix. The fastest modes (with larger eigenvalues λ) are more localized and have steeper energy walls with a larger decrease in entropy. They are, therefore, referred to as kinetically hot residues. For more details, see the Supplementary Materials and [56].

The connection between kinetically hot residues and interface residues has been established already [60]. The methodology described here moves forward and introduces a self-adjusting approach that is aimed at recognizing binding surfaces and corresponding structural scaffolds (contact and neighboring first-layer residues; that approach was initially given in [61,62]). The results depicted here show that at least one of the proteins that forms a heterodimer has its contacting scaffold surrounded or bounded through its kinetically hot residues. One of the partners (usually the longer one) has binding areas and corresponding binding scaffolds defined by its kinetically hot residues, while its partner is presumably more flexible. It may pass through structural adjustments, which means that the recognition of its binding residues could be difficult with the coarse-grained methodology based on the distribution of kinetically hot residues only. A similar difficulty is encountered in heterodimers composed of similarly sized proteins (with similar chain lengths). Furthermore, with smaller proteins, the adjustable GNM approach may be less precise, because the small protein size easily produces many false positives. However, the fact that at least one of the binding partners has binding areas defined by its kinetically hot residues (and thus is less movable than other residues) may suggest that the heterodimer protein formation is entropically driven, i.e., that the protein chains often interact in an attempt to increase the overall entropy (i.e., increase the entropy of rigid binding scaffolds).

The term “kinetically hot residues” is similar to the term “hot spots” that is often used in protein science, but these terms have much more in common than linguistics. Residues that often appear in structurally preserved interfaces (in more than 50% of cases) are termed hot spots. The hot spots are important, because they are general contributors to the binding free energy. They are screened using the alanine-scanning mutagenesis and are therefore defined as spots where alanine mutation increases the binding free energy at least 2.0 kcal/mol [63,64,65,66,67,68,69]. Bogan and Thorn [63] showed that hot spot residues are enriched in tryptophan, tyrosine, and arginine, and that they are surrounded with residues whose role is to occlude solvent from the hot spots (O-ring residues hypothesis). They also observed that “*(n)either the change in total side-chain solvent-accessible surface area on complex formation (ΔASA) nor the sidechain ΔASA of hydrophobic atoms is well correlated to the change in free energy*” [63]. They concluded that solvent occlusion is a necessary but not sufficient condition for a residue to be a hot spot. The hot spots have been addressed using various computational methods [65]. Tuncbag et al. used information on conservation, the solvent accessibility area, and the statistical pairwise residue potentials of the interface residues to computationally determine hot spots. Their combined approach achieved both accuracy and precision between 64% and 73% of the Alanine Scanning Energetics and Binding Interface Databases. They observed that “*conservation does not have significant effect in hot spot prediction as a single feature*”. However, their results indicate that the “*residue occlusions from solvent and pairwise potentials are found to be the main discriminative features in hot spot prediction*”. Lise et al. [66,67] combined machine learning and energy-based methods to predict hot spot residues. They applied standard energy terms (Van der Waals potentials, solvation energy, hydrogen bonds, and Coulomb electrostatics) as input features to Support Vector Machine (SVM) and Gaussian Processes learning protocols. They also attempted to predict the change in binding free energy *ΔΔG* upon alanine substitution but achieved only a limited success. Den et al. [70] also used Support Vector Machines with Random Forest selection and Sequential Backward feature elimination to predict hot spots. They used various molecular attributes (local structural entropy, side chain energy score, four-body pseudo-potential, weighted relative surface area burial) as feature vector elements, as well as the residue neighborhood defined via the Euclidian distances between residues/heavy atoms, with Voronoi diagram/Delaunay triangulation employed to describe residue’s neighbors. They ended with 38 features, which the SVM protocol utilized to predict hot spots very efficiently. Kozakov et al. [68] analyzed druggable hot-spots via a computational method that places small organic molecules—probes (16 of them)—on a grid around target protein. The spots on the surface of the target protein that favorably interact with a number of probes are clustered, and those clusters are ranked according to the average free energy. The consensus regions (the regions that bind many probes) are taken to be hot spots. Their method is able to recognize hot spots even in unbound cases. The authors concluded that according to their protocol, the hot spots “*possess a general tendency to bind organic compounds with a variety of structures, including key side chains of the partner protein*”. This sentence is emphasized, because the results depicted in this paper also show that the binding interface of large host proteins is often determined by their own structure only. That may imply that host proteins are receptive for other proteins and/or small molecules besides their usually encountered binding partners. The method depicted here is also able to distinguish dimer decoys that were created with structures of unbound chains (see Testing set analysis in this paper). Similarly, Tuncbag et al. [69] observed that “*globally different protein structures can interact via similar architectural motifs*”. They employed that fact through the PRISM algorithm that “*utilizes rigid-body structural comparisons of target proteins to known template protein-protein interfaces and flexible refinement using a docking energy function.*” 

To develop a really useful prediction method for a biological system as demonstrated in a series of recent publications (see, e.g., [71,72,73,74,75,76,77,78,79,80,81,82,83,84,85]), and especially in a set of publications relevant to the topic of protein-protein, protein-ligand, and protein-drug interactions [37,38,39,40,41,42,43], one should observe and possibly follow the 5-step methodology [86]; (I) how to construct or select a valid benchmark dataset to train and test the predictor; (II) how to formulate a set of biological sequences or structure samples with an effective mathematical expression that can truly reflect an intrinsic correlation with the targets to be predicted; (III) how to introduce or develop a powerful algorithm (or engine) to operate the prediction; (IV) how to properly perform cross-validation tests to objectively evaluate the anticipated accuracy of the predictor; (V) how to establish a user-friendly web-server for the predictor that is accessible to the public. 

This paper follows the above-described 5-step methodology. It starts with an overview of methods and tools (a short overview of the theoretical background of the Gaussian network model is given in the Supplementary Materials). The definition of target residues, as well as the short description of training and testing sets, is given after that. The first simple prediction protocol that is based on the five fastest modes and the sequential influence of the hot residues only is given in the third chapter. The same chapter describes a prediction approach that uses the modes that correspond to the upper 10% of the eigenvalues range. After that, the paper offers a brief description of the behavior of dimers with different sequence lengths of their protein constituents and introduces a significant improvement in the prediction based on the adjustable number of fast modes. After that, the paper describes an adjustable prediction protocol based on the 3D influence of hot residues, as well as the combination of sequential and spatial approaches. Finally, the paper describes an evaluation of adjustable protocols on the Sternberg [87] and Vakser decoy sets [88]. While doing so, the paper compares the adjustable GNM to the Lu and Skolnick’s detailed, residue-level statistical potential approach to contact residues recognition [89]. The paper ends with the Conclusion. 

## 2. Materials and Methods 

### 2.1. GNM Code

The software for the Adjustable Gaussian Network Model code is composed of several different programs. The first program calculates contact maps and the corresponding eigenvectors and eigenvalues [90] for both protein chains that form a protein dimer (given as a PDB file). To accomplish that, the program first calculates the Kirchhoff contact matrix **Γ** for each protein. The matrix **Γ** calculation is based on the distances between C_α_ atoms only, and those distances have to be less than or equal to 7 Å to consider two residues in a contact [54,55,56]. The code then calculates and sorts **Γ** matrix eigenvalues and eigenvectors. The eigenvectors are sorted according to their corresponding eigenvalues. Those eigenvalues and eigenvectors are used in the second part that (iteratively) calculates the weighted sum of modes [57] as

(1) $$\langle {\left(\Delta R_{i}\right)}^{2}\rangle {}_{k_{1}-k_{2}}=\left(k_{B}T/\gamma \right)\overset{k_{2}}{\underset{k_{1}}{\sum }}\lambda_{k}^{-1}{\left[u_{k}\right]}_{i}^{2}/\overset{k_{2}}{\underset{k_{1}}{\sum }}\lambda_{k}^{-1}$$

This equation, normalized by dividing the sum by , produces mean square fluctuations of each residue by a given set of modes (*k*_1_ to *k*_2_). The equation produces an estimate of a kinetic contribution of each residue for a given set of modes. The above equation is very similar to the singular value decomposition method [91] used in the linear least squares optimization method. For details on the phantom networks and the Gaussian Network Model, see a short overview in the Supplementary Materials. An additional code extracts contact and first-layer residues. Finally, the third set of routines extracts neighboring residues and their distances for each residue per protein chain. That information is later used in the spatial spreading of the influence of kinetically hot residues. 

### 2.2. Targets

The aim of the methods presented here is to recognize contact patches on protein surfaces and the corresponding scaffolds in the protein interiors. The first aim is to recognize contact residues. These are amino acid residues in which at least one atom is at the maximum distance of 4.5 Å from one or more atoms from the surface of the other chain. The distance of 4.5 Å corresponds to the size of one water molecule. The second aim is to recognize the first-layer residues (FLR), i.e., residues in contact with contact patches, but which are not contacts themselves. Therefore, those are neighboring residues from the same protein (at the maximum atom-atom distance of 4.5 Å from contact residues). They form scaffolds that surround contact residues (for a visual description of contact and first-layer residues, see Figure S1 in the Supplementary Materials). 

### 2.3. Training Set

The training set is comprised of 433 protein dimer complexes (see Supplementary Materials for the full list of dimers; this set is inspired by the Chen dimer set). It is separated into heterodimer and homodimers, using two criteria: (1) If the ratio of protein lengths (protein sequence length is the number of its amino acid residues) in a dimer complex is greater than 2, that complex is considered to be heterodimer; (2) If the ratio of protein lengths is smaller than 2, the Smith-Waterman sequence alignment algorithm [92] is applied to recognize and separate dimers in which proteins sequences are highly similar. This approach was applied following the homology modeling principle that says that high sequence similarity implies structural similarity [93,94,95,96]. In some cases, proteins were considered to be heterodimers, although they have a high sequence similarity due to large sequence gaps (1IAI and 1EKI). Therefore, the first group contains dimers in which constituents do not bear obvious structural similarity, while the second group has members that are sequentially and structurally highly similar. The dimers were separated into two groups, because heterodimers and homodimers may exhibit different binding mechanisms. Different behaviors of these two groups may imply that their kinetically hot residues may not be have the same role in protein binding. This approach was used because the Gaussian Network Model is based on structural organization of residues, i.e., on the spatial distribution of C_α_ atoms in proteins. 

Of 433 dimers, 139 are heterodimers, and the rest are homodimers. Majority of proteins in our training set have sequences shorter than 300 residues, but we also have a number of proteins longer than 400 residues. The distribution of chain lengths is shown in Figure S2 in the Supplementary Materials.

### 2.4. Testing Sets

The Sternberg [87] and Vakser [88] decoy sets are numerically created decoy sets created for testing and evaluation of protein binding prediction protocols. Each decoy set is based on a naturally occurring protein dimer complex with a known structure. Each individual decoy from a set is a protein complex numerically created by joining two (or more) individual proteins based on the corresponding non-bound structures. 

The Sternberg decoys sets [87] are comprised of 100 decoys each with first four being near native structures and the first one being the native structure itself. The decoys are generated using unbound structures of the chains that form native dimer structures. In this work only dimer sets were used (10 sets). The adjustable GNM algorithms were applied independently to both proteins per decoy. 

Every Vakser set contains 110 decoys. Certain number of those decoys are near native structures (in most cases, 10 of decoys in a set are near native, as determined by their root mean square deviations from the native structure(s)). Only dimer sets (41 of the 61 decoy sets) were used in this research. 

## 3. Results and Discussion

### 3.1. Simplest 1D Prediction (Sequential Neighbors Influence only) Based on 5 Fastest Modes

The first attempted method is based on the approach of Demirel et al. [57], which used five fastest modes to recognize kinetically hot residues in proteins. With a direct implementation of their scheme, the first step was the calculation of the weighted sum (Equation (1)). With normalized sum, only residues with the normalized amplitude higher than 0.05 (hot residues) were tested against extracted contact and first-layer residues. The number of hot residues is usually smaller than the number of contact or first-layer residues. To account for that, the influence of hot residues was spread to their sequential neighbors using sequence information obtained from their structure PDB files (to account for possible missing residues). The influence of hot residues was spread linearly, to sequential neighbors only, because proteins are polymer chains with physically connected residues. That implies that sequentially neighboring residues should exhibit correlated behavior. For chains longer than 100 amino acids, hot residues and 8 their sequential neighbors upstream and downstream were labeled as predictions (four upstream, four downstream). For shorter chains, the influence was spread to 6 neighboring residues only. The labeled residues are assumed to be either contact or first-layer residues. This approach was used on all 433 dimers regardless of the sequence length or the nature (hetero or homodimer) of a particular dimer complex. 

**Figure 1:**
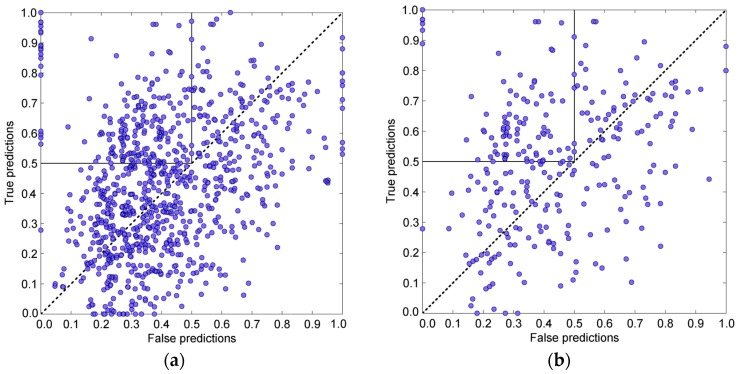

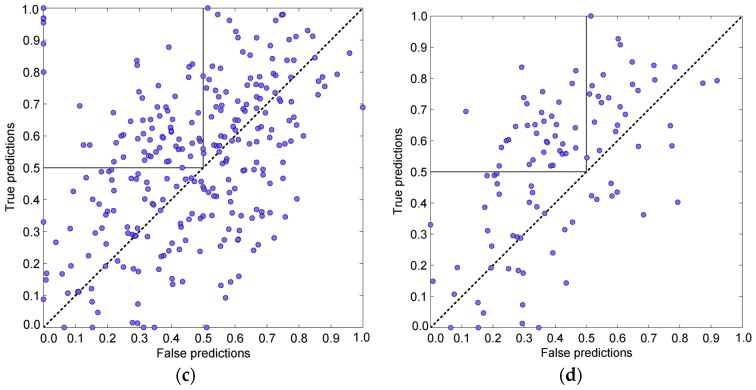
(**a**) Prediction output (ratio of true vs. false predictions depicted as a scatterplot) for a simple prediction approach based on the 5 fastest modes, for each protein chain in our list (433 dimers in total). The diagonal line separates area where true positives outpace false positives from the area where false positives are dominant. The square in the upper left quadrant is the area of good predictions (ratio of true predictions is greater than 0.5, and ratio of bad predictions is lass or equal than 0.5). The true positives mean is 43.09%, and the false positives mean is 40.77%. There is 22.17% of good predictions (192 chains, they are in the upper left quadrant) and 12.70% of very bad predictions (110 chains). The very bad predictions are in the lower right quadrant, which is not depicted as rectangle to emphasize the importance of good predictions. (**b**) Prediction output for 278 heterodimers chains only, for the basic approach based on the 5 fastest modes. The true positives mean is 50.74%, and the false positives mean is 42.68%. There is 31.29% of good predictions (87 chains) and 11.15% of very bad predictions (31 chains). (**c**) Prediction output for the simple approach based on the fastest 10% of modes per chain for all heterodimers (278 chains). The true positives mean is 52.52%, and the false positives mean is 46.27%. There is 23.02% of good predictions (64 chains) and 14.39% of very bad predictions (40 chains). (**d**) Prediction output for the simple approach based on the modes that correspond to top 10% of the eigenvalues range, for heterodimer chains with high sequence length ratios (the chain length ratio >2, the individual chain lengths longer than 80 residues). The true positives mean is 52.03%, and the false positives mean is 40.67%. There is 33.01% of good predictions (34 chains) and 6.80% of very bad predictions (7 chains).

Figure 1a shows the algorithm output for all 866 proteins (433 dimers). The ratio (percentage) of true predictions versus ratio of false prediction per protein is depicted on a two dimensional Cartesian plane, i.e., as a scatterplot. The ratio of true predictions per protein is the number of true predictions over the total number of targets (contact and first-layer residues). They are true positives. The ratio of false predictions per protein is the number of residues falsely predicted as being either contact or FLR over the total number of non-target residues, and they treated as false positives. The Cartesian plane is separated into two parts by a diagonal going from the lover left to the upper right quadrant. The proteins above the diagonal are considered as satisfying prediction, because the ratio of their true positives over false positives is over 1. The proteins under the diagonal are, obviously, unsatisfying, i.e., they are considered bad predictions. The chains (i.e., predictions) in the upper left quadrant we define as good predictions (the ratio of true positives is above 0.5, and the ratio of false positives lower or equal to 0.5). In addition, very bad predictions are taken to be the ones that fall into the lower left quadrant (the ratio of false positives is over 0.5, and the ratio of true positives lower or equal than 0.5). Henceforth, these two measures, percentage of good predictions and percentage of bad predictions, besides true positives mean, and false positives mean, will be used as measures of the quality of the prediction methods. There are obviously better definitions of good and bad prediction. The two used in this research were applied primarily for the algorithm tuning, because they are easy to interpret and implement. 

Figure 1a clearly shows that satisfying and unsatisfying predictions are almost equally distributed. The true and false means are 43.09% and 40.78%, respectively. The percentage of good predictions (22.17%) is higher than the percentage of very bad predictions (12.70%), but the amount of good predictions is still not good enough for the general purpose application. However, the distribution of good and bad predictions is not uniform over the protein chain lengths, as the histogram in Figure S3 in the Supplementary Materials nicely depicts. The prediction method based on the five fastest modes is much more successful with shorter (and thus less voluminous) proteins than with longer ones. With proteins longer than 100 residues, but shorter than 200, the prediction algorithm was not satisfying at all, because it put more predictions in the lower right quadrant than in upper left. However, with proteins shorter than 100 residues, it put much more predictions in the upper left (good predictions) than in lower right quadrant, which means that 5 modes may be only good for smaller proteins. 

To test the assumption that heterodimers behave differently from homodimers, the above-described method was applied on heterodimers only (278 chains). Figure 1b depicts the results of that analysis. It is obvious that more predictions are in the upper left quadrant than in the lower right. That indicates that hot residues and their neighbors, recognized using only five fastest modes, are much closer to binding patches on the surface and in the interior of heterodimer chains. On average, there are 50.74% of true positives and 42.68% of false positives. The distribution of good and very bad predictions is better than with the complete set (see Figure S4 in Supplementary Materials) but is still not satisfactory enough, because there is only 31.29% of good predictions (87 chains in the upper left quadrant) and 11.15% of bad ones (31 proteins are in the lower right quadrant).

Figure S5 in the Supplementary Materials depicts the example of this initial approach on four different protein chains. It shows the weighted sums for those four proteins, their contact and first later residues (expressed as the ratio of atoms per the total number of atoms in residue), and the predictions. It is clearly visible that, for the longer proteins (chain P from 1BVN in particular), five fastest modes fail at predicting the target residues. For shorter chains (2SNI chain E, 1UDI chain E, and 1CXZ chain A), five modes are better at connecting the kinetically hot residues to contact and FLR patches, but the overall prediction is still not very favorable, because the percent of the accurately predicted contact and FLR residues is comparatively small.

### 3.2. First Attempt to Improve the Prediction

#### 3.2.1. Prediction Based on the Fast Modes That Corresponds to Top 10% of the Eigenvalues

The previous analysis and the corresponding distribution of good and bad predictions over the protein sequence lengths indicate that the weighted sum based on five modes only is not able to decipher the contact patterns on the surface and in the interior of proteins. With shorter protein chains (up to 100 residues), the weighted sum of five fastest modes is able to give a satisfying prediction of binding and first-layer residues, but with longer chains, the prediction efficiency fails (Figures S3 and S4 in the Supplementary Materials). Therefore, it can be assumed that the number of modes should be adapted to each individual protein. That number would be difficult to determine knowing only the length of a protein chain, because, when sorted, the distribution of the mode intensities (eigenvalues) is not a linear function of the mode index (see Figure S6 in the Supplementary Materials). The distribution of eigenvalues depends on the chain’s length, as well as on the protein’s three-dimensional configuration. Therefore, a new approach with a variable number of modes that corresponds to the top 10% of the eigenvalues span for every protein was attempted. Figure S6 nicely depicts that top ten percent of eigenvalues are covered by five modes for the protein 1ETT chain H, itself made of 231 amino acid residues. The protein chain P from dimer 1BVN (496 residues) has 14 modes covering top 10%, and the protein chain A from 1QGK (876 residues) has 29 fastest modes covering the top 10% of eigenvalues. There is a correlation between the number of residues and the number of fast modes, but it is not strictly linear.

When this approach is applied to heterodimers, the amount of true positives becomes increased. That can be observed with the four protein chains analyzed previously (Figure S7 in the Supplementary Materials). However, the percentage of false positives also gets increased. With the whole set of heterodimers (278 protein chains), the overall improvement is miniscule, the true positives mean is 52.52%, and the false positives mean of 46.27% (Figure 1c). The increase of the false positives mean corresponds to the decrease of the number of good predictions to 23.02% of the total number of proteins (64 proteins), as well as the increase of the very bad prediction to 14.39% (40). The distribution of good and very bad predictions over the chain lengths shows that this approach is not ideal for all protein chains (Figure S8 in the Supplementary Materials). The bad predictions are dominant for proteins longer than 100 residues and shorter than 200 residues. However, this approach is able to accurately access contact and first-layer residues in a protein with very high number of residues (876, chain A from 1QGK).

#### 3.2.2. Analysis of Heterodimers with Very Different Sequence Lengths

Heterodimers are protein complexes composed of two protein chains with no apparent sequential and structural similarity. What forms such entities? What kind of attraction forces two or more different protein chains to from a stable structure? In protein-DNA or protein-lipid interactions, electrostatic forces are often the key binding factors, but with protein interactions such forces usually have a small or negligible influence. 

When a protein dimer is analyzed, one may wonder whether its two constituents evolved separately, or whether they created by a mutation that broke a single protein chain into two separate parts. If we expand this premise, we can assume that such mutation can more easily survive if a point of separation is close to the terminal ends of the initial, single chain (single mutation is, of course, a euphemism for a much more complex random biological process). In that case, a longer sub-chain has a higher probability of preserving its fold and function, because it will be highly homologous to the initial chain (homology implies similar folding patterns, see [93,94,95,96]). The probability of surviving is much higher than with mutations that break a protein into constituents of similar sizes. Namely, a protein produced by an asymmetric breaking will more easily preserve its fold and have more of a chance of surviving evolutionary pressures. That may also imply that a protein with longer sequence (more voluminous protein), produced by that single mutation, when interacting with its shorter partner (if that partner survived throughout evolution), may preserve its fold during (and upon) the binding. Similarly, if dimer constituents evolved separately, longer partners may be less prone to significant structural changes during the binding due to their sheer size. All this may imply that kinetically hot residues may determine the shape and the position of a scaffold that determines binding spots in individual heterodimer chains. 

To test this assumption, the previously analyzed heterodimers were divided into two groups according to the length ratios of their constituents. The heterodimers with sequence length ratios higher than two were analyzed separately from the rest of heterodimers. Figure S9 in the Supplementary Materials depicts the analysis of the heterodimer chain lengths. The panel (a) depicts the sequence length for each monomer, with longer monomers given via the green line and shorter via the blue line. The panel (b) depicts the corresponding sequence length ratios. The vertical line separates heterodimers into heterodimers with sequence length ratios higher than two from heterodimers with smaller sequence length ratios. The chains with sequence lengths shorter than 80 were eliminated from this group to reduce the occurrence of chains with high percentage of both true and false positives.

Figure 1d depicts the results of the analysis of heterodimers with high sequence length ratios of constituents. In the analysis, only modes that corresponded to top 10% of eigenvalues range were used. This approach, although based on a smaller subset of proteins, shows a visible improvement. It is obvious that the number of proteins with badly characterized target residues (proteins in which the ratio of true positives vs. false positives is less than 1) is reduced. The true positive mean is 52.03%, and the false positive mean is 40.67%. Although only 6.80% of predictions are characterized as very bad (7 proteins), the method is still not satisfactory, because only 33.01% of all chains (34 proteins) are in the upper left quadrant (good predictions). The distribution of good vs. very bad predictions (Figure S10 in the Supplementary Materials) shows much better behavior of this prediction method over the protein sequence lengths than the previous two attempts.

The same analysis, performed on proteins forming heterodimers with low sequence length ratios (for proteins with more than 80 residues), reveals a different picture (Figure S11 in the Supplementary Materials). There is only 13.64% of good predictions (18 proteins out of 132) versus 20.45% of very bad predictions (27 proteins). The true positives mean is 52.75%, a value very similar to the true negatives mean of 53.18%. The distribution of good vs. very bad predictions (Figure S12 in Supplementary Materials) is also not very favorable to good predictions and indicates a negative correlation between kinetically hot residues and binding scaffolds in heterodimers of similar size.

### 3.3. Prediction Based on the Adjustable Number of Modes

The previous attempts to recognize contact and first-layer residues via the Gaussian Network Model were based on a static approach in which protein dimer structures were analyzed using either 5 fastest normal modes or modes that corresponded to the top 10% of the eigenvalues range. Those approaches showed that kinetically hot residues may play a role in protein-protein interactions, but they did not offer enough proof for that assertion. With some protein chains they produced excellent results, but with some they failed. More importantly, the percentage of good predictions (the amount of chains with more than 50% of true positives and less than 50% of false positives) was comparatively small (always less than 40% of all the chains analyzed). Many of the proteins had a very high percentage of both true positives and false positives. In addition, a significant number of proteins had a very small percentage of both true and false predictions. All of this implied that prediction algorithm had to be improved.

**Figure 2:**
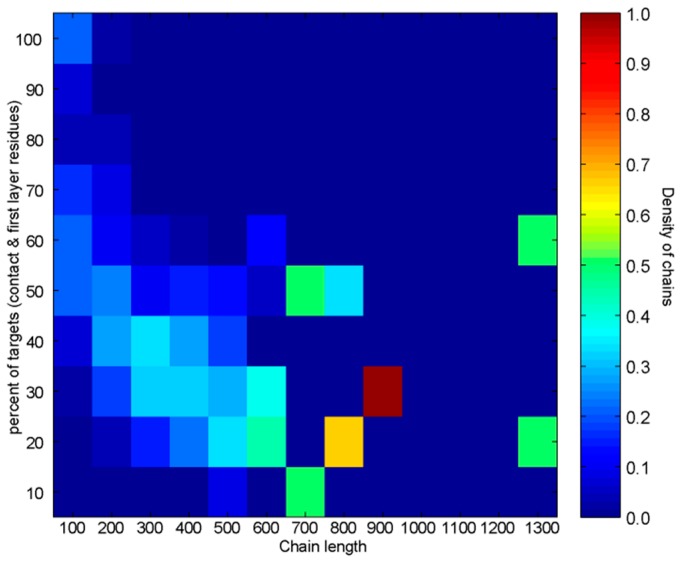
Distribution of targets per sequence length for 414 dimers that belong to the training set depicted as a heat map. The burgundy square designates a length/percent pair with a highest concentration of chains. Yellow and light green squares are length/percent pairs with a medium number of chains. The dark blue squares are length/percent pairs with low occupancy. The navy areas designate zero chain occupancy. It is obvious that the percent of targets is a decreasing function of the sequence length.

The analysis of the average percentage of targets per sequence length reveals that the amount of targets and the chain length are inversely proportional. Larger proteins with longer sequences have a smaller percentage of contact and first-layer residues than shorter chains. Figure 2 depicts the distribution of targets over the protein sequence lengths. It clearly shows that small proteins (shorter sequence lengths) have much higher ratio of contact and first-layer residues than larger proteins (longer amino acid sequence lengths). 

The information on the targets distribution can be used to improve the prediction approach. The prediction can be adjusted to each particular protein chain through a comparison of the current prediction output, i.e., current ratio of predictions (the total number of residues assigned to be either contact or first-layer residues by the algorithm) over the total number of residues, to the expected, i.e., average, percentage of targets for that protein’s sequence length class. The improvement of the prediction algorithm can be performed as follows:

– If the overall percentage of predictions is too large for that protein’s sequence length class (for example, if the percentage of predictions is larger than 60% of the total number of residues), the number of fast modes should be reduced by one, and the whole prediction procedure should be repeated (Equation (1)).
– If the percentage of predictions is too small for the protein’s sequence length class (e.g., less than 20% of all residues), the number of fast modes should be increased by one, and the whole prediction procedure should be repeated (Equation (1)).
– The procedure should be repeated until the percentage of predictions does not fit between the maximum and minimum amount of predictions for a given sequence length.

#### 3.3.1. One-Dimensional Linear Prediction

A simple strategy adjusts the number of modes for each particular chain: if the number of residues in a chain is less than or equal to 300, too many predictions are taken to be 60%. In that case, i.e., if the amount of predictions is over 60% of all residues, the number of modes is reduced by one, and the prediction procedure is repeated (Equation (1)). Similarly, if the number of residues is greater than 300, too many predictions are taken to be 50%. Furthermore, if the chain length is less or equal to 500 residues, too few predictions are taken to be 40%. For cases like that, the number of modes is increased by one, and the whole procedure is repeated. For longer chains, too few predictions are 20%. To avoid infinite loops, only one increase followed by a decrease is allowed, and vice versa. The prediction procedure itself spreads the influence of kinetically hot residues linearly upstream and downstream along the sequence, as with the previously described methods. The procedure starts with a number of modes that correspond to the top 10% of eigenvalues range for the protein being analyzed. This approach ensures that longer proteins have enough predictions, and that shorter ones are not saturated with too many false positives. 

**Figure 3:**
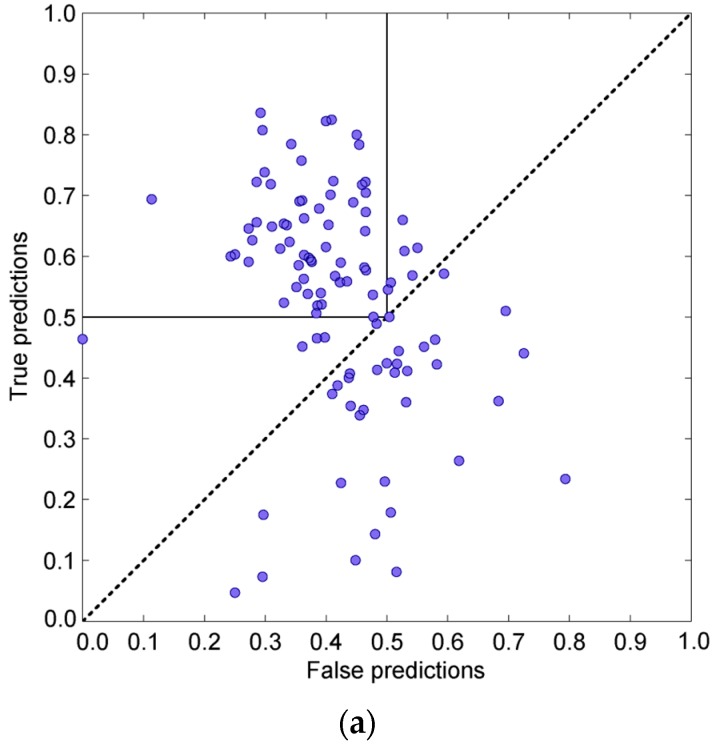

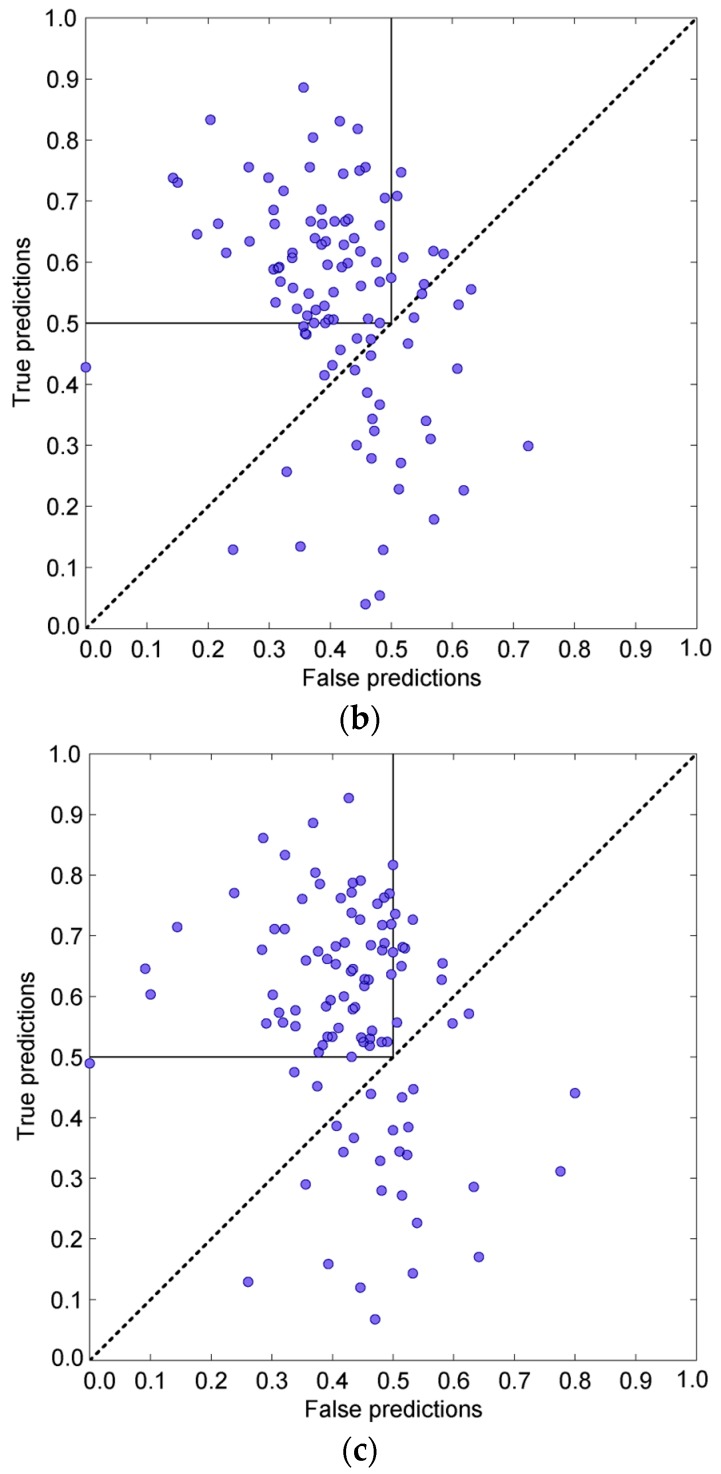
(**a**) Algorithm output for the prediction based on the adjustable number of fastest modes per chain and sequential influence of hot residues, for high sequence-length ratio dimer chains (length ratio greater than two, chain length greater than 80 residues). The true positives mean true is 53.27%, and the false positives mean is 42.05%. There is 56.31% of good predictions (58 of 103 chains) and only 14.56% of very bad predictions (15 chains). (**b**) Algorithm output for the prediction based on the adjustable number of fastest modes per chain and the variable 3D influence per hot residue (the influence of a hot residue is spread to spatial neighbors closer than 6 or 8 Å), for chains in dimers with high sequence length ratios (Length ratio > 2, length > 80 residues). The true positives mean true is 53.77%, and false positives mean is 41.29%. There is 56.31% of good predictions (58 chains) and 8.74% of very bad predictions (9 chains). (**c**) Algorithm output for the prediction based on the adjustable number of fastest modes per chain and combined 1D & 3D influences of hot residues, for chains in dimers with high sequence length ratio (Length ratio > 2, length > 80 residues). The true positives mean is 56.77%, and the false positives mean is 43.21%. There is 63.11% of good predictions (65 chains) and 11.65% of very bad predictions (12 chains).

Figure 3a shows how this adaptable approach works with heterodimers with high sequence length ratios (the length ratio larger than two and chain lengths longer than 80). The statistical analysis shows a remarkable improvement over the previous prediction attempts. The true positives mean is 53.27%, and the false positives mean is 42.05%. There is 56.31% of good predictions (58 proteins) and only 14.56% of very bad predictions (15 proteins). The distribution of good and very bad predictions over the chain lengths is also very favorable (Figure S13 in the Supplementary Materials).

The analysis performed on the four chains used previously to describe the prediction procedure confirms the above results (Figure S14 in the Supplementary Materials). For the three longest protein chains, 1BVN chain P, 2SNI chain E, and 1UDI chain E, the percent of true positives is over 50%, and the percent of false positives is less than 50%. The protein chain E of 1UDI has a highest difference between the true and false positives, which is an indication of a high correlation between the kinetically hot residues and contact scaffolds for that chain. Only the shortest example, 1CXZ chain A, has both true and false positives over 50%. 

When this approach is applied to heterodimer proteins with low sequence length ratios (with the chains longer than 80 residues), the results are less than satisfactory (Figure S15 in the Supplementary Materials). The true positives mean is 50.39%, and the false positives mean is 49.53%. The amount of good and bed predictions is very close, namely, there is 34.85% of good predictions (upper left quadrant, 46 of 132 proteins) and 27.27% of very bad predictions (lower left quadrant, 36 proteins). The distribution of good and bad predictions is also not favorable (Figure S16 in the Supplementary Materials). 

#### 3.3.2. 3D Spatial Prediction—Variable Influence of Hot Residues

The adjustable algorithm introduced in the previous chapter uses sequential neighbors only to spread the influence of hot residues. It produces good predictions of contact and first-layer residues but offers a room for improvement. The prediction can be improved if spatial neighbors, instead of sequential ones, are used to spread the influence of hot residues. This approach is much closer to the true nature of the GNM algorithm that uses only spatial distances between C_α_ atoms and disregards any sequential/connectivity information. To apply this approach, the maximum cutoff C_α_-C_α_ distance from the center of a hot residue was introduced, from which its influence can be spread. The cutoff distance of 6 Å was applied with for shorter protein chains (for sequence lengths shorter than 250), and the cutoff of 8 Å was applied for longer protein chains. All residues that are within the sphere centered at the C_α_ atom of the hot residue and within the assigned cutoff distance are considered to be “predictions”, i.e., they are assumed to be either contact or first-layer residues. All other residues are rejected (for that particular hot residue). The two cutoff values were estimated empirically. To extract spatial neighbors, distances between residues (C_α_-C_α_ distances) were calculated for each particular protein and sorted in ascending order. 

Figure 3b depicts the algorithm output for the heterodimers with the sequence length ratios higher than two for protein chains with a chain length longer than 80 residues. The true positives mean is 53.77%, and false positives mean is 41.29%. There is 56.31% of good predictions (58 proteins) and only 8.74% of very bad predictions (8 proteins). There is also a noticeable number of predictions with a very favorable ratio of true positives vs. false positives, which are outside the upper left quadrant and thus do not belong to the good predictions as we defined them. Figure S17 in the Supplementary Materials shows the distribution of good and very bad predictions. Figure S18 in the Supplementary Materials shows the predictions for the four examples used previously.

With proteins from low sequence length ratio dimers, the results are not as good. The true positives mean is 52.22%, and the false positives mean is 48.81%. There is 42.42% of good predictions (56 proteins) and 27.27% of very bad predictions (36 proteins). See Figures S19 and S20 in the Supplementary Materials.

#### 3.3.3. Combining the Sequential and Spatial Approaches

The two methods described previously base their prediction on the adjustable number of modes. The first method spreads the influence of hot residues linearly, i.e., to sequential neighbors only, while the second method spreads the influence to spatial neighbors within a sphere of a given cutoff radius. The first method treats a protein chain as a set of amino acids that are chained together. The second method only sees the spatial-3D neighborhood of a hot residue and rejects the fact that the protein is an ordered set of amino acids that are physically connected. This chapter introduces the combination of the sequential and 3D spatial approaches in attempt to boost the overall prediction. By combining the one-dimensional linear approach with the three-dimensional one, the residue connectivity information is included into the structure based method, which thus takes into account the chain-like nature of proteins (GNM method disregards chain connectivity and uses only physical distances between C_α_ atoms to calculate the protein connectivity matrix). In this combined approach, the influence of a hot residue is first spread linearly to its sequential neighbors (upstream and downstream along the sequence). After that, the influence is spread to the hot residue’s spatial neighbors whose C_α_ atoms are within a sphere of a given cutoff radius with a center in the hot residue’s C_α_ atom (the radius is 6 or 8 Å, depending on the sequence length). 

Figure 3c shows the effects of the combined approach. When applied to the set of heterodimers with a high sequence length ratio, this approach produces an increase in the true positives mean (56.77%) without a significant increase in the false positives mean (43.21%). More importantly, the combined approach puts 63.11% of proteins in the upper left quadrant (good predictions, 65 proteins), but keeps very bad predictions at a reasonably low 11.65% (12 proteins). The number of good predictions for sequence lengths between 200 and 300 is slightly increased, as well as the number of predictions for sequence lengths between 400 and 500 (see Figure S21 in the Supplementary Materials). This change may indicate that method based on the variable influence of hot residues on their spatial neighborhood works better with longer protein chains. That may be expected, because bigger proteins with longer sequences have more modes and offer finer resolution with the weighted sum than smaller proteins with shorter sequences. See also Figure S22 for the four examples used previously.

With the proteins from low sequence-length ratio dimers, the situation is, as expected, not as good. The true positives mean is 51.57% to the false positives of 50.00%. There is 37.88% of predictions in the upper left quadrant (50 out of 132 proteins) to 29.55% in the lower right quadrant (39 proteins), see Figures S23 and S24 in the Supplementary Materials.

**Figure 4:**
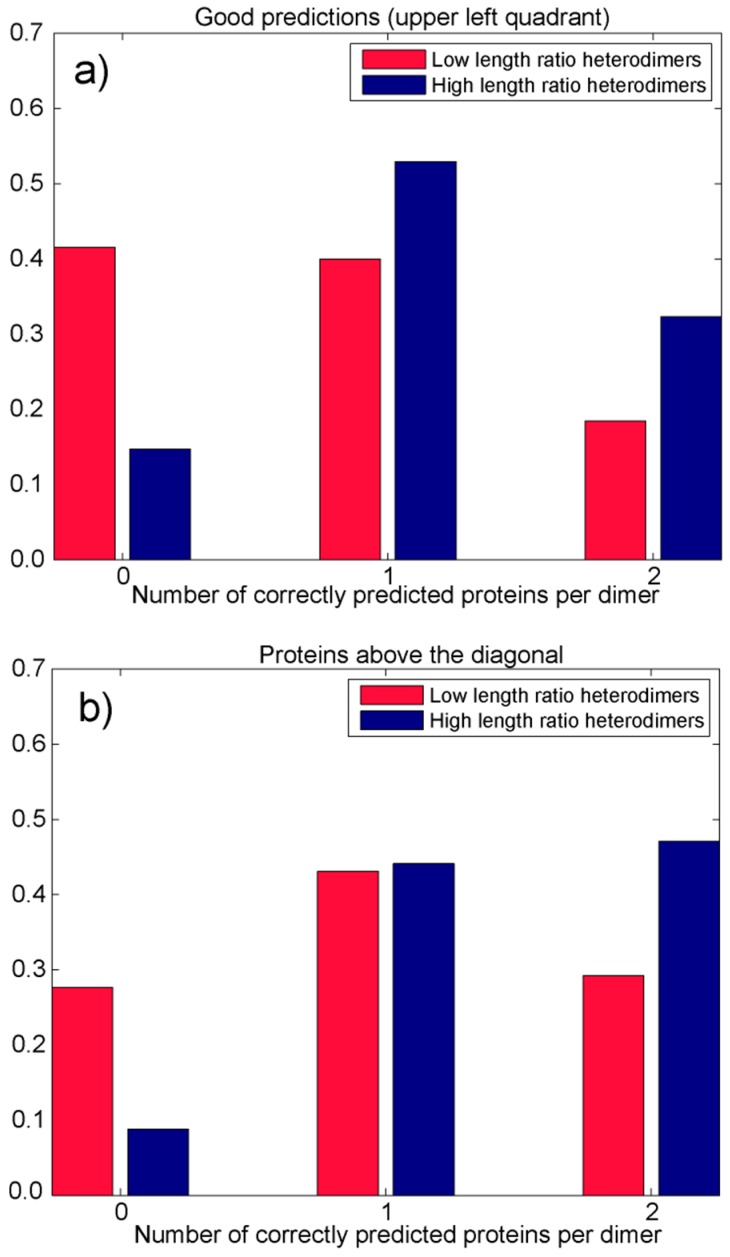
Number of correctly predicted chains per heterodimer using the combined (1D and 3D) adjustable approach, for dimers in which both chains are longer than 80 residues. Two cases are analyzed: heterodimers with long sequence length ratios (>2) and heterodimers with short sequence length ratio (≤2). (**a**) Number of chains per dimer in the upper left quadrant. (**b**) Number of chains per heterodimer above the main diagonal (the diagoanal that passes through the lower left and upper right quadrants).

When both proteins per dimer are addressed as a pair using the combined approach (adjustable GNM, plus 1D & 3D influence of kinetically hot residues), the analysis confirms that heterodimers with high sequence length ratios often behave quite differently from heterodimers with low sequence length ratios (see Figure 4). In majority of cases belonging to the former group, at least one protein has contact and first-layer residues gathered around its kinetically hot residues (as recognized by the adjustable GNM). Figure 4a shows that 85.29% of the high sequence length ratio dimers has at least one chain in the upper left quadrant (32.35% of those dimers has both chains in the upper left quadrant, and 53% only one chain), as opposed to 58.46% of the low sequence length dimers (only 18.46% of them have both chains in the upper left quadrant). Only 8.82% of the high sequence length ratio dimers has none of the chains above the diagonal, as opposed to 27.69% of the low sequence length dimers (Figure 4b). In 47% of cases, the high sequence length ratio dimers have both chains above the diagonal (44.12% of high sequence dimers has only one). On the other hand, 29.23% of the low sequence length ratio dimers have both chains above the diagonal (43.08% of low sequence heterodimers has only one chain above the diagonal). This analysis suggests that proteins that form high sequence-length ratio heterodimers (sequence length ration higher than 2) often behave like a rigid lock and key. They have at least one rigid interfacial surface (often both surfaces are rigid). Chains forming low sequence-length ratio dimers are presumably more flexible in that respect and more often than not have one of the chains more flexible than the other. That protein chain adjusts its conformations for a tighter fit. This should not be a general conclusion because smaller (sequentially shorter) protein chains have a large number of false positives, simply because they have relatively large total number of targets (contact and first-layer residues, see Figure 2). A similar observation was made by Martin and Lavery [97]. They concluded that the surface of a small chain easily gets saturated with contacts when bound to a larger partner. With larger proteins, contact residues are highly localized. They also observed that docking hits tend to accumulate closer to the geometrical center of the protein. That observation is in concordance with the approach presented here, which, besides contact residues, also uses first-layer residues to enhance the prediction. Residues in the geometrical center are surrounded with a large number of neighbors and have higher packing density. They are, therefore, more stable and thus emphasized by the fast modes.

### 3.4. Prediction Algorithms Comparison

**Figure 5:**
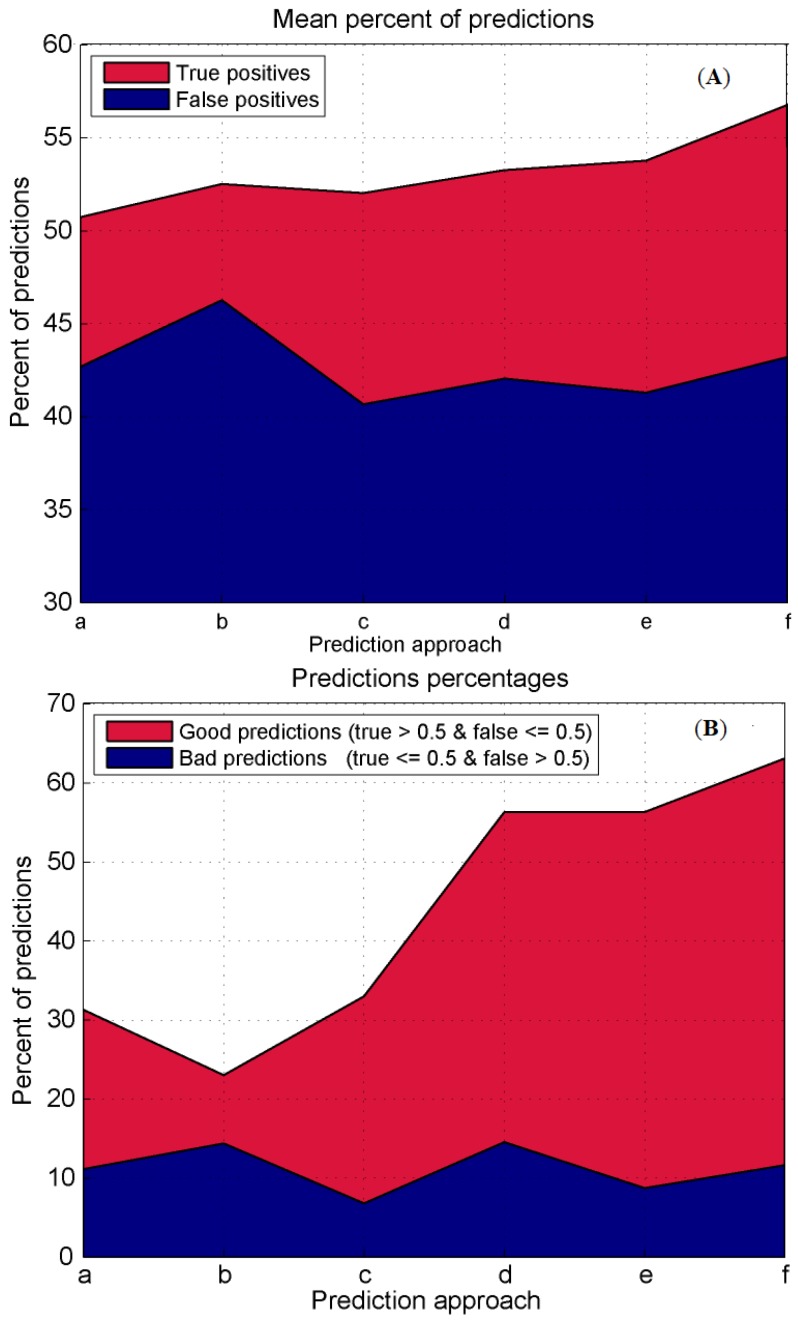
(**A**) Prediction algorithms comparison expressed as a plot of the true positives mean and the false positives mean percentages for each algorithm described previously. The first two algorithms were applied on all heterodimer chains. In all other cases, algorithms were applied on the heterodimer chains with high sequence-length ratios. The algorithms are (a) all heterodimers, 5 fastest modes; (b) all heterodimers, fastest modes corresponding to top 10% of eigenvalues range; (c) high sequence length ratio, fastest modes corresponding to top 10% of eigenvalues range; (d) adjustable number of modes, 1D influence; (e) adjustable modes, 3D influence, within a sphere with a radius of 6 or 8 Å; and (f) algorithms **d** and **e** combined. (**B**) Prediction algorithms comparison expressed as a percentage of good and very bad chains. The first two algorithms were applied on all heterodimer chains. In all other cases, algorithms were applied on the chains with high sequence-length ratios. The algorithms are (a) all heterodimers, 5 fastest modes; (b) all heterodimers, with fastest modes corresponding to top 10% of eigenvalues range; (c) high sequence length ratio, with fastest modes corresponding to top 10% of eigenvalues range; (d) adjustable number of modes, 1D influence; (e) adjustable modes, 3D influence, within a sphere with a radius of 6 or 8 Å; (f) algorithms **d** and **e** combined.

In the previous chapters a number of methods for the contact and first-layer residues prediction were presented. The presentation started with a very simple approach based on the fixed number of modes (5) and ended with a protocol that adjusts the number of modes to the analyzed chain and spreads the influence of a hot residue in the adaptable fashion using the sequential and spatial influence of hot residues. The true evaluation of these protocols can be done only through a direct comparison of their prediction efficiencies. The comparison of the true positives mean vs. the false positives mean and comparison of their good vs. very bad predictions are obvious measures of the quality of the prediction, and we used them here (see Figure 5). As the figure shows, the true prediction improvement is achieved only with the adjustable number of modes (prediction protocol **d** in the Figure 5a,b). Additional improvements are achieved with the full 3D influence spread (protocol **e** in Figure 5a,b), as well as with the combination of sequential (1D) and spatial (3D) approaches (protocol **f** in Figure 5a,b).

**Figure 6:**
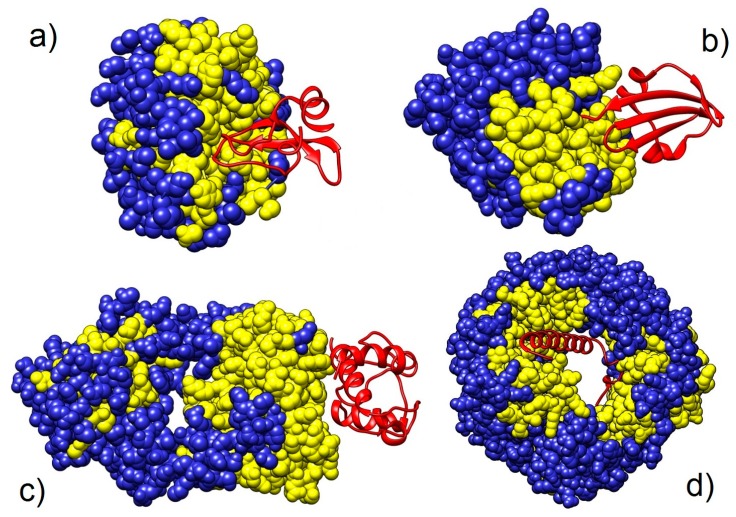
Ability of the adjustable 1D&3D GNM algorithm to predict binding scaffolds. It is depicted via four heterodimers (PDB ID codes 1BRC, 1DTD, 1WEJ, and 1QGK). The analyzed chains are blue, depicted using the whole atom representation, with the adjustable GNM predictions colored yellow. Partnering chains are red and depicted as ribbons. (**a**) Chains E and I from the protein 1BRC. The chain E was analyzed with the adjustable GNM. This is a very good prediction. There is 75.29% true positives, with 47.41% false positives. (**b**) Chains A and B from the protein 1DTD. The chain A was analyzed with the adjustable GNM. This is a very good prediction. There is 71.08% true positives, and only 30.45% false positives. For the chain A, only residues 363 to 665 are given in the PDB file. There is a Zinc atom and four water molecules embedded in the interface (not shown). The binding interface is defined only using the weighted sum (Equation (1)). (**c**) Chains L and F from the protein 1WEJ. The chain L was analyzed with the adjustable GNM. This is a very good prediction. There is 92.73% true positives, and 42.67% false positives. (**d**) Chains A and B from 1QGK. The chain A was analyzed with the adjustable GNM. This is a very good prediction. There is 88.58% true positives, and only 36.83% false positives.

Figure 6 illustrates the ability of the adjustable approach (1D and 3D influences combined) to recognize binding scaffolds. It nicely depicts than in some cases, the adjustable GNM very accurately predicts the binding scaffolds.

The analysis of the relationship between the number of modes and sequence length (Table S1 and Figure S25 in the Supplementary Materials) reveals an interesting trend. For protein chains shorter than 600 residues, with accurately predicted contact and first-layer residues via the combined approach, the relationship between the number of fast modes *n* and the sequence length *s* is roughly linear (*m* = 2.1831 + 0.014254 × *s*, i.e., *m* ≈ 2.1831 + (1/70) × *s*). However, when longer chains are included, the relationship becomes quadratic (*m* = 3.4794 + (0.00030756) × *s* + (2.8381 × 10^-5^) × *s*^2^). Those relationships are strongly influenced by the distribution of chains in the training set (Figure S1 in the Supplementary Materials). A more uniform distribution will probably change the shapes/slopes of these two lines. It should be noted that two relationships are close to each other for chains shorter than 600 residues; Figure S25 in the Supplementary Materials nicely depicts those trends. Haliloglu et al. [60] used a cutoff of 15% of the number of residues to establish a number of modes used in kinetically hot residues recognition. Our results show that the number of fast modes is generally smaller.

### 3.5. Vakser and Sternberg Decoy Sets

Previous chapters dealt with the development of the contact residues recognition protocol. This chapter depicts how the adjustable protocol behaves with the Vakser [88] and Sternberg decoy sets [87]. Those decoy sets are numerically created protein structure sets created with the intention of evaluating the quality of protein binding prediction protocols. To properly evaluate the adjustable GNM protocols, for each decoy in both sets, contact and first-layer residues were calculated for each protein that formed a dimer. All adjustable GNM algorithms were tested, and the 3D adjustable approach showed the best overall results. 

For each decoy pair, the binding energy was calculated using the statistical potential of Lu and Skolnick [89]. That energy was used to compare and evaluate the adjustable GNM prediction protocol against a residue level statistical potential. The residue-residue based approach of Lu and Skolnick assesses the strength of each decoy (taking both chains together) using an empirical statistical potential (given as a 20 × 20 matrix). The binding affinity of each decoy is expressed as a potential energy of binding. The lower that energy is, the more probable the decoy is, according to the statistical potential method. 

**Figure 7:**
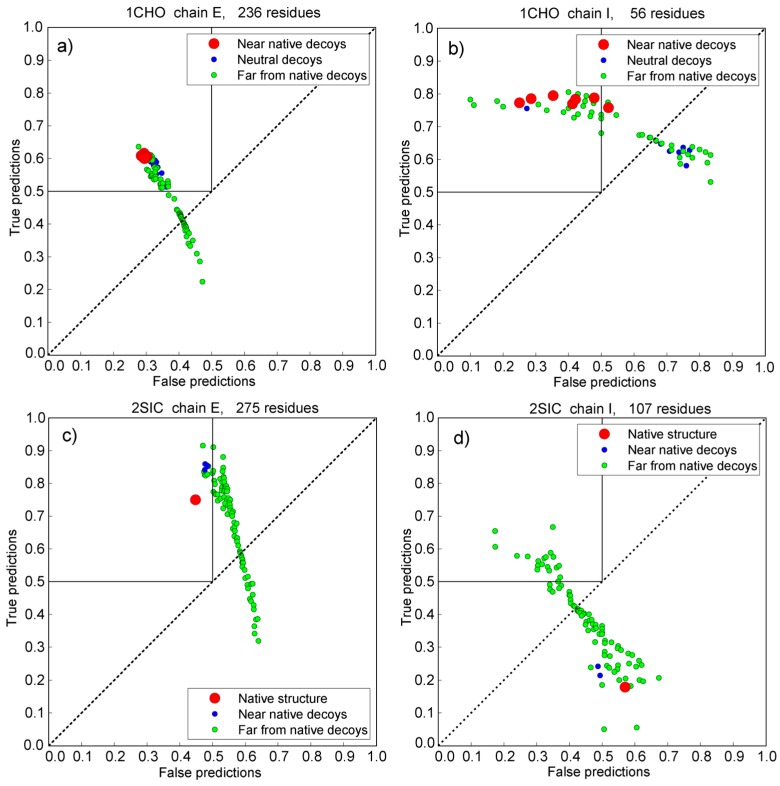
Protein dimer decoys recognition using the adjustable GNM protocol. The influence of hot residues is spread to spatial neighbors closer than 6 or 8 Å. Subplots (**a**) and (**b**) are from the Vakser decoy sets (PDB ID 1CHO). Blue circles depict neutral decoys regardless (neither native nor far from it). Decoys far from native structure are green, and near native ones are red. Subplots (**c**) and (**d**) are from the Sternberg decoy sets (PDB ID 2SIC). Green dots depict far from native decoys. Near native decoys are blue, and the native structure is a red circle.

**Figure 8:**
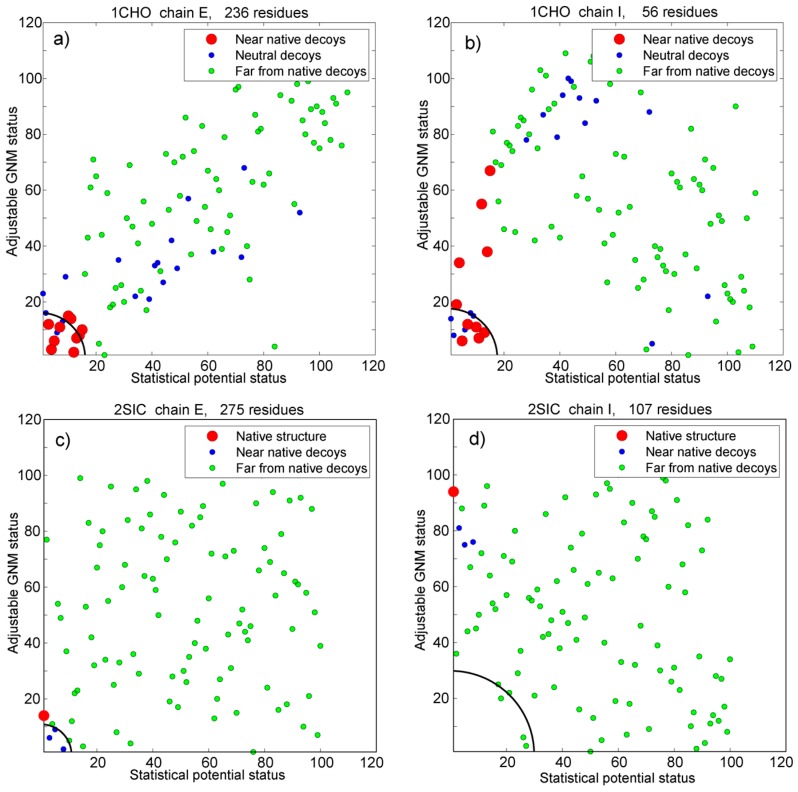
Comparison of the abilities of the adjustable spatial GNM approach and the statistical potential to distinguish near native decoys/structures from false decoys. Blue dots depict neutral decoys. Decoys far from native structure are green, and near native ones are red. Two decoys sets are depicted (1CHO from Vakser set, and 2SIC from Sternberg set), with two chains per example. The left plot in each example corresponds to the longer chain, and the right plot to its shorter pair. The circular segments in the lower left corners correspond to the distances of the *n*-th best chain according to the combined approach of the adjustable GNM and the statistical potential, in which *n* is the number of near native structures. It is a good measure of the concordance between the two methods.

Figure 7 depicts the behavior of decoys on the true/false scatter plot used in previous chapters via two decoy subsets (1CHO and 2SIC). The standing of each protein chain is calculated as its Cartesian distance from the point with coordinates (0, 1), i.e., the standing of a protein is its “distance” from a point with 0% of false predictions and 100% true predictions. Figure 8 depicts the behavior of the adjustable GNM in combination with the statistical potential using the same two decoy subsets.

The quality of the assessment of both methods (adjustable GNM and statistical potential) is expressed via two scores: the best status of a near native decoy among the all decoys, and the coverage, i.e., the number of near native decoys among the best *n* decoys, in which *n* is the number of near native decoys. Those two evaluations are depicted in Tables S2 and S3, and in Figures S26 and S27 in the Supplementary Materials. The combination of the two approaches is given in the last 6 columns of the Tables S2 and S3. The combined standing is given as a Cartesian distance of a chain with two scores between 1 and 110 (100 for Vakser) from the point with coordinates (1, 1). The point (1, 1) corresponds to a structure that should be first according to the both methods.

Our analysis reveals that in 19 out of 41 Vakser decoys sets (1AVW_AV, 1BUI_AC, 1BVN_PT, 1CHO_EI, 1EWY_AC, 1FM9_DA, 1GPQ_DA, 1HE1_CA, 1MA9_AB, 1OPH_AB, 1PPF_EI, 1UGH_EI, 1WQ1_GR, 1YVB_AI, 2BKR_AB, 2FI4_EI, 2SNI_EI, 3SIC_EI, 2BTF_AP), and in 4 out of 10 Sternberg decoys sets (1BRC, 1UGH, 1WQ1, 2SIC), either one or both chains are properly accessed by the adjustable GNM method. Those observations are even more significant if decoy sets badly characterized by both methods (adjustable GNM and statistical potential) are removed from the analysis (Vakser sets 1F6M_AC, 1G6V_AK, 1GPQ_DA, 1TX6_AI, and Sternberg set 1AVZ). The chains that form these dimers probably experience significant structural rearrangements during or upon the binding. In most cases, the longer chain is better assessed through the adjustable GNM than its shorter partner, which was to be expected following the assumption of the opposite behavior of binding partners, but in some cases (Vakser sets 1BVN_PT, 1GPQ_DA, 1HE1_CA, 1MA9_AB, 2BKR_AB, 2BTF_AP) the shorter partner has a higher score. The adjustable GNM protocol is fairly successful in predicting near native structures. That information should be taken in the light of fact that near native decoys are based on nonbound structures, which makes the protocol even more successful. Similar ability was reported by Kozakov et al. [68]. The statistical potential produces much better overall results, but in some cases (Vakser set 1PPF_EI, for example) the structural evaluation of the decoys set was better than the evaluation using the empirical statistical potential.

## 4. Conclusions

This paper addresses the physical interactions of proteins, which is an important issue in molecular biology and biophysics. It depicts a method based on the theory of phantom networks [48,49,52] and its Gaussian Network Model expansion [54,55,56,57,60]. The described methodology attempts to relate the stable, kinetically hot residues, together with residues in their direct neighborhood (together termed binding scaffolds), to the binding residues on the surface and in the interior of the proteins forming protein dimers. As the number of residues emphasized by GNM is usually smaller than the number of target residues, an improvement of GNM was developed to spread the influence of each kinetically hot residue to its neighbors in an adjustable fashion. The paper first describes a method based on a small and fixed number of fast modes and a similar approach that uses the fast modes that correspond to the upper 10% of the eigenvalues span. Both approaches offer only a limited ability to correlate the kinetically hot residues and the binding scaffolds. A limited improvement was achieved with heterodimers with a significant difference in chain length. The true improvement was produced when the number of modes was allowed to fluctuate until the number of predictions matched the expected number of targets for a given sequence length. The combination of sequential and spatial influence was shown to have the best ability to recognize the target residues. That may imply that connectivity information, although not explicit in the Gaussian Network Model, partially determines the kinetic behavior of residues. Therefore, it should be used together with the graph representation of protein structure in the analysis of the behavior of proteins and their contact patterns. 

The adjustable GNM protocols were tested on Sternberg [87] and Vakser [88] decoy sets. With both sets, the adjustable GNM approach with the spatial spread only achieved a noticeable success in predicting target residues. The combined approach using both the adjustable GNM and the statistical potential of Lu and Skolnick [89] improved the prediction in comparison to the pure adjustable approach. 

The approach described here can be an excellent guide to a more efficient drug design, especially for the design of small molecule inhibitors of protein-protein interactions [44,45,46,47]. The algorithm(s) depicted here can help in filtering “in-house” databases [45] and thus facilitate the drug screening process. 

As pointed out in [98] and demonstrated in a series of recent publications (see, e.g., [71,72,73,75,79,82,83,85,99,100,101,102]), user-friendly and publicly accessible web-servers represent the future direction for developing more useful and practical prediction methods. Actually, many practically useful web-servers have had an increasing impact on medical science [103], driving medicinal chemistry into an unprecedented revolution. We shall make efforts in our future work to provide a web-server for the prediction method presented in this paper.

The adjustable approach, although able to dynamically connect the kinetically hot residues and binding patches and the corresponding structural scaffolds, still has a space for improvement. For instance, the application of surface area descriptors may reduce the false positives rate. A better estimation of the number of expected target residues may also improve the prediction. The application of the latest protein-protein and protein-ligand databases [46] may also help in that regard.

This work was primarily focused on the behavior of heterodimers. Homodimers were not explored in detail. Their behavior should be addressed more thoroughly, for example, by using a combination of slow and fast modes [30]. Furthermore, the slow modes describe global motions of chain segments and may lead toward a better understanding of the conformational changes that proteins undergo during and upon binding. Those changes still lack proper quantification [104].

The importance of this work is twofold. First, it gives an efficient algorithm that is able to decipher individual protein-protein interactions, and it offers a theoretical insight into the mechanism of protein binding. Second, it shows that a simple approach based only on the statistic of residue-residue interactions may lead to overfitting; thus, the shape of partnering protein chains, as well as the absolute and relative size of the interacting proteins (molecules), should be also taken into account. 

The fact that in heterodimers, at least one of the proteins has its binding scaffold determined by the kinetically hot residues may imply that protein-protein interactions are, at least partially, entropically driven [105]. Highly organized pockets delineated by kinetically hot residues attract physically smaller partnering proteins in an attempt to increase the total entropy of the system (i.e., to decrease the structural order defined by unmovable, kinetically hot residues). This observation opens an area for further research. 

## Supplementary Materials

The following are available online at http://www.mdpi.com/1424-8247/11/1/29/s1, Figure S1: An illustration of the targets, Figure S2: Protein chain lengths distribution for both heterodimers and homodimers, Figure S3: Prediction histogram based on the analysis of all chains over the sequence lengths for the simple prediction approach based on five fastest modes, Figure S4: Prediction histogram for heterodimer chains only, for the simple prediction approach based on the 5 fastest modes, Figure S5: An example of the one dimensional, i.e., sequential approach to prediction, for 4 different chains (1BVN chain P, 2SNI chain E, 1UDI chain E and 1CXZ chain A), Figure S6: Distributions of eigenvalues for three different protein chains (dimer 1ETT chains H, dimer 1BVN chain P and dimer 1QGK chain A), Figure S7: An example of the 1D prediction (sequential neighbors influence only) based on the fastest 10% of modes per chain, for 4 different chains (1BVN chain P, 2SNI chain E, 1UDI chain E and 1CXZ chain A), Figure S8: Prediction histogram for heterodimer chains only, for the prediction approach based on the modes that correspond to top 10% of the eigenvalues span, Figure S9: Dimer chain lengths for the set of 139 different heterodimers, Figure S10: Prediction histogram, for chains in heterodimers with high sequence length ratios for the prediction approach based on the modes which correspond to top 10% of eigenvalues span, Figure S11: Prediction output for chains in heterodimers with low sequence length ratios for the prediction approach based on modes corresponding to top 10% of eigenvalues range, Figure S12: Prediction histogram over the sequence lengths for the simple prediction approach based on the fastest 10% of modes for each chain, for chains in heterodimers with low sequence length ratios, Figure S13: Histogram of predictions over the sequence lengths for the prediction approach based on the adjustable number of fast modes, with the 1D influence of hot residues, for chains in dimers with high sequence length ratio, Figure S14: Examples of the prediction based on the adjustable number of fast modes and the sequential influence of hot residues, Figure S15: Prediction output based on the approach that uses an adjustable number of fastest modes per chain and sequential influence of hot residues, for low sequence-length ratio dimer chains, Figure S16: Prediction histogram over the sequence lengths for the prediction approach based on the adjustable number of fast modes, for the 1D influence of hot residues, for chains in dimers with low sequence length ratio, Figure S17: Prediction histogram over the sequence lengths for the prediction approach based on the adjustable number of fast modes and variable 3D influence per hot residue, for chains in dimers with high sequence length ratio, Figure S18: Examples of the prediction based on the adjustable number of fast modes and the sequential influence of hot residues, Figure S19: Prediction output for the prediction approach based on the adjustable number of fastest modes per chain and the variable 3D influence per hot residue, for chains in dimers with low sequence length ratios, Figure S20: Prediction histogram over the sequence lengths for the prediction approach based on the adjustable number of fast modes and the variable 3D influence per hot residue, for chains in dimers with low sequence length ratio, Figure S21: Prediction histogram over the sequence lengths for the prediction approach based on the adjustable number of fast modes and combined 1D & fixed 3D influence per hot residue for chains in dimers with high sequence length ratio (length ratio higher than 2, length > 80 residues), Figure S22: Examples of the prediction based on the adjustable number of fast modes and combined 1D & 3D influence per hot residue, Figure S23: Prediction output for the prediction approach based on the adjustable number of fastest modes per chain and combined 1D & 3D influences of hot residues, for chains in dimers with low sequence length ratio, Figure S24: Prediction histogram over the sequence lengths for the prediction approach based on the adjustable number of fast modes and combined 1D & fixed 3D influence per hot residue for chains in dimers with low sequence length ratio, Figure S25: Linear and quadratic relationships of the number of modes per chain, for successfully characterized heterodimer chains from dimers with high sequence length ratios, Figure S26: Comparison of the abilities of the adjustable 3D GNM approach, the statistical potential and their combination to distinguish near native decoys from the false decoys, Figure S27, Comparison of the abilities of the adjustable 3D GNM approach, the statistical potential and their combination to distinguish near native decoys from the false decoys, Table S1: The summary of good predictions, Table S2: The efficiency of the adjustable prediction algorithm (3D algorithm with variable number of modes) with the Vakser decoy sets, Table S3: The efficiency of the adjustable prediction algorithm (3D algorithm with variable number of modes) with the Sternberg decoy sets.
