# Supplementary material for: Heterodimer Binding Scaffolds Recognition via the Analysis of Kinetically Hot Residues
Source: Pharmaceuticals (Basel). 2018 Mar 16;11(1):29. doi: 10.3390/ph11010029 (PMC5874725; doi:10.3390/ph11010029)
Supplement: Supplementary file 1 [file pharmaceuticals-11-00029-s001.pdf]

Heterodimer binding scaffolds recognition via the analysis of kinetically hot residues

Supplementary material

Ognjen Perišić[1,2]

March 13th 2018

[1] Big Blue Genomics
Vojvode Brane 32,
11000 Belgrade, Serbia
[2] New York University,
Department of Chemistry,
1001 Silver, 100 Washington Square East
New York, New York 10003, USA
Email: ognjen.perisic@gmail.com

Gaussian Network Model Theory

Biological polymers can be perceived as canonical ensembles (NVT ensembles described by the number of particles N, volume V and temperature T). That implies that they should be treated as systems in statistical equilibrium that do not evolve over time. They are also in thermal equilibrium with each other. Two proteins brought into contact will retain the same ensembles; their combined ensemble will be canonical ensemble itself. They are guided by the Boltzmann distribution, i.e., molecular bonds can be seen as independent entities guided by the Boltzmann distribution (the probability of bond *i* having the energy E_i is $p_i = e^{-E_i/k_B T}$, with a partition function $Z = \sum_i e^{-E_i/k_B T}$). Further approximation treats polymers as phantom networks.

The theory of phantom networks was introduced by James and Guth in 1947 [48]. It was further expanded by Flory [49, 52]. The phantom network theory assumes that: (a) the mean values $\bar{r}$ of the individual chain vectors are linear functions of the tensor λ of the principal extension ratios specifying the macroscopic strain (they are affine in strain); (b) fluctuations $\Delta r = r - \bar{r}$ about the mean values are Gaussian, and (c) the mean square fluctuations depend only on the structure of the network and not on the strain [49, 52].

The theory of phantom networks begins with the assumption that chains and junctions can move freely through each other. It is also assumed the equilibrium Gaussian distribution of polymer constituents as a density distribution $W(r)$

$$W(r) = \left(3/2\pi\langle r^2\rangle_0\right)^{3/2} \exp\left(-3r^2/2\langle r^2\rangle_0\right), \tag{S1}$$

W(r) can be expressed as $W(r) = \tilde{Z}_r / Z$. Z is the configurational integral for the free chain, and $\tilde{Z}$ is the configurational integral over the configurational space in which is *r* is restricted to a given value [52].

The configuration partition function Z_N for the network may be written as a product of the partition functions of the network's *v* individual chains. The individual partition functions are fully determined by the end-to-end vectors r_{ij} that connect junctions *i* and *j* [49, 52]. Therefore, Z_N can be expressed as a product of all junction *ij* connected by a chain as [49, 52]

$$Z_N = \prod_{i<j} \tilde{Z}_{rij} = Z^v \prod_{i<j} W(r_{ij}). \tag{S2}$$

The partition function Z_N can be described by the end-to-end vectors r_{ij} as

$$Z_N = C\prod_{i<j} \exp\left(-3r_{ij}^2 / 2\left\langle r_{ij}^2 \right\rangle_0\right). \tag{S3}$$

The sum of these vectors can be expressed through the distances R_i between junctions

$$Z_N = C \cdot \exp\left(-\frac{1}{2}\sum_i \sum_j \gamma_{ij}^* \left|R_i - R_j\right|^2\right), \tag{S4}$$

with coefficient γ_{ij} being equal to $\gamma_{ij}^* = 3/2\left\langle r_{ij}^2 \right\rangle_0$ if junctions i and j are connected by a chain, zero otherwise [49, 52].

The sum of end-to-end vectors in the Eq. S4 can be expressed via the quadratic symmetric matrix $\mathbf{\Gamma}$ [49, 52]

$$\sum_{i<j} r_{ij}^2 = \frac{1}{2}\sum_i \sum_j \left|R_i - R_j\right|^2 = \{R\}^T \mathbf{\Gamma}\{R\}. \tag{S5}$$

This transformation can be easily proved via the basic tools of linear algebra.
The elements of the matrix $\mathbf{\Gamma}$ are

$$\mathbf{\Gamma} = \begin{cases} \gamma_{ij} = -\gamma_{ij}^*, & i \neq j \\ \gamma_{ii} = \sum_j \gamma_{ij}^*, & i = j \end{cases} \cdot \tag{S6}$$

If all non-zero elements γ_{ij} are equal, and that is the case when all chain links are equal, consequently all $\left\langle r_{ij} \right\rangle_0$ identical and matrix $\mathbf{\Gamma}$ **is** Kirchhoff contact matrix [49, 52]. Therefore, Eq. S4 can be written as

$$Z_N = C \cdot \exp\left(-\{R\}^T \mathbf{\Gamma}\{R\}\right). \tag{S7}$$

If one of the junctions is designated as zeroth, then all others can be measured from that one. In polymers we have two types of junctions. The matrix $\mathbf{\Gamma}$ can be represented as a composition of two sets of junctions, fixed junctions σ that usually give shape to the phantom network, and free junctions τ. The sum in the above partition function of the phantom network can be decomposed as [49, 52]

$$\{R\}^T \Gamma\{R\} = \{R_\sigma\}^T \Gamma_\sigma \{R_\sigma\} + 2\{R_\tau\}^T \Gamma_{\tau\sigma}\{R_\sigma\} + \{R_\tau\}^T \Gamma_\tau \{R_\tau\}. \tag{S8}$$

In this equation Γ_σ is the quadratic matrix composed of rows and columns of matrix $\mathbf{\Gamma}$ for the fixed junctions. Γ_τ is the corresponding matrix for the free junctions, and $\Gamma_{\tau\sigma}$ is the rectangular matrix composed of the rows from the set {τ} and columns from the set {σ}.

Eq. S8 can be further simplified, by separating the free junctions τ and the fixed junctions σ as [52]

$$\{R\}^T \Gamma \{R\} = \{R_\sigma\}^T G_\sigma \{R_\sigma\} + \{\Delta R_\tau\}^T \Gamma_\tau \{\Delta R_\tau\}, \quad \text{(S9)}$$

where

$$G_\sigma = \Gamma_\sigma - \Gamma_{\sigma\tau}\Gamma_\tau^{-1}\Gamma_{\tau\sigma} \quad \text{(S10)}$$

$$\{\Delta R_\tau\} = \{R_\tau\} - \{\overline{R}_\tau\}, \quad \text{(S11)}$$

with

$$\{\overline{R}_\tau\} = -\Gamma_\tau^{-1}\Gamma_{\tau\sigma}\{R_\sigma\}. \quad \text{(S12)}$$

$\{\overline{R}_\tau\}$, within this framework [52], define the most probable positions for the free junctions.

The partition function of the phantom network (Eq. S7) thus can be written as

$$Z_N = C \cdot \exp\left(-\{R_\sigma\}^T G_\sigma \{R_\sigma\} - \{\Delta R_\tau\}^T \Gamma_\tau \{\Delta R_\tau\}\right). \quad \text{(S13)}$$

This function is a multivariate normal distribution. The integration of this function over the free junctions produces a form that does not depend on free junctions at all [52]

$$Z_{N,\sigma} = C\pi^{\frac{3}{2}n\tau} \det(\Gamma_\tau)^{-3/2} \exp\left(-\{R_\sigma\}^T G_\sigma \{R_\sigma\}\right). \quad \text{(S14)}$$

In 1997 Haliloglu, Bahar and Erman [54, 55] applied the above-described theory of phantom networks to folded proteins and thus introduced the Gaussian Network Model (GNM). They removed fixed junctions σ following the assumption that the protein folding is not guided by the external constraints. In their approach the contact matrix $\mathbf{\Gamma}$ was calculated with the cutoff distance of 7 Angstroms, i.e., the residues are in contact only if their C_α - C_α distance is less or equal than 7 Å [56, 57, 58]. They also used the approximation of M. Tirion [46] which replaces non-bonded interactions with Hookean springs, and

defines γ* to be constant. In their approach the Kirchhoff contact matrix **Γ** is defined via Heaviside's step function [56] as

$$\mathbf{\Gamma} = \begin{cases} -H(r_c - r_{ij}) & i \neq j \\ \sum_{i(\neq j)}^{N} \Gamma_{ij} & i = j \end{cases} \tag{S15}$$

Therefore, the diagonal elements of the matrix **Γ** in this approximation represent local packing densities around the residues in the protein. In the native state, in equilibrium, protein assumes stable conformation with minimum energy in respect to all residue fluctuations (the protein is a canonical ensemble) [56]. The vibrational contribution to the Helmholtz free energy is [56]

$$A = -k_B T \ln Z_N = -(3k_B T/2)\ln\left[(\pi/\gamma^*)^{N-1}\det(\mathbf{\Gamma}^{-1})\right]. \tag{S16}$$

The partition function Z_N is the vibrational partition function given by $Z_N = \int \exp\{-H/k_B T\}d\{\Delta R\}$, and γ* is $\gamma/2k_BT$. The last equality in Eq. S16, originally derived by Flory [52], comes from the integration of the single parameter multivariate Gaussian function in the configurational integral. Therefore, the internal Hamiltonian of the protein, $H = \frac{1}{2}\gamma\left[\Delta R^T \cdot \mathbf{\Gamma} \cdot \Delta R\right]$ is expressed via the contact matrix **Γ** [52]. Within the GNM framework, ΔR are fluctuations of C_α atoms around their most probable positions [56].

The average Hamiltonian can be expressed in terms of the matrices U and Λ of the eigenvectors u_i and eigenvalues λ_i of the matrix **Γ** as [56]

$$\langle H \rangle = \frac{1}{2}\gamma\langle \Delta R^T U \Lambda U^T \Delta R \rangle = \frac{1}{2}\gamma\sum_{i=2}^{N}\lambda_i\langle \Delta r_i^2 \rangle = \frac{3}{2}(N-1)k_B T, \tag{S17}$$

because every symmetric (square) matrix, such as the contact matrix **Γ**, can be transformed into a canonical form via its eigenvalues Λ and eigenvectors U. The last two equalities in Eq. S17 stem from the fact that $\langle \Delta r_i^2 \rangle$ are diagonal elements of the correlation matrix $\langle \Delta r \cdot \Delta r^T \rangle = U^T \langle \Delta R \Delta R^T \rangle U = U^T \langle (3k_B T/\gamma)\mathbf{\Gamma}^{-1} \rangle U = (3k_B T/\gamma)\Lambda^{-1}$. The correlation of equilibrium fluctuations of two α carbons i and j, can be expressed as [54]

$$\langle \Delta R_i \cdot \Delta R_j \rangle = (k_B T/\gamma)[\Gamma^{-1}]_{ij} \tag{S18}$$

The average Hamiltonian in the formulation of the Helmholtz free energy $A = \langle H \rangle - TS$, is thus expressed via the fluctuations of C_α atoms (residues) fluctuations in mode space, $\Delta r_i = U^T \Delta R_i$ [56]. Eigenvectors U in this framework can be interpreted as fluctuation modes of C_α atoms and eigenvalues Λ as their corresponding mode intensities. Slow, large amplitude modes, with small λ_i, correspond to polymer's (protein's) global motions, while fast, small amplitude modes, with large λ_i, correspond to polymer's (protein's) localized motions (hot residues) [56]. Therefore, residues having high amplitude fast mode fluctuations are stable – unmovable. My aim is to decipher the role of those kinetically hot residues. That can be accomplished by combining individual residue contributions into the weighted sum [57] as

$$\langle (\Delta \mathbf{R}_i)^2 \rangle_{k_1 - k_2} = (k_B T/\gamma) \sum_{k_1}^{k_2} \lambda_k^{-1} [\mathbf{u}_k]_i^2 / \sum_{k_1}^{k_2} \lambda_k^{-1}. \tag{S19}$$

This equation, normalized by diving the sum by $(3k_B T / \gamma)$ gives mean square fluctuations of each residue by a given set of modes (k_1 to k_2) sorted by their corresponding eigenvalues. In this paper, fastest modes are used, with the upper bound k_2 being equal to the number of modes, i.e. number of residues, and k_1 being variable. The above equation is very similar to the singular value decomposition method [91] used in the linear least squares optimization method.

The correspondence of GNM to real world experimental values was confirmed by showing that the vibrational spectrums obtained by GNM strongly correlates to crystallographic B factors [57, 58, 59] and NMR data [59], which means that equilibrium fluctuations are properties of static crystals.

Training dimer set list:

104L, 11BA, 11BG, 11GS, 137L, 14GS, 15C8, 167L, 16GS, 174L, 175L, 176L, 17GS, 18GS, 19GS, 19HC, 1A03, 1A04, 1A05, 1A0A, 1A0E, 1A0F, 1A0G, 1A0M, 1A0O, 1A10, 1A19, 1A1U, 1A22, 1A25, 1A28, 1A2L, 1A2M, 1A2O, 1A2X, 1A3L, 1A3Y, 1A4F, 1A4I, 1A4R, 1A4U, 1A4X, 1A50, 1A5C, 1A5F, 1A64, 1A6D, 1A6E, 1A6J, 1A6P, 1A6U, 1A6W, 1A71, 1A73, 1A74, 1A75, 1A78, 1A7A, 1A7H, 1A7N, 1A7O, 1A7P, 1A7Q, 1A7R, 1A7V, 1A7X, 1A8J, 1A8T, 1A8V, 1A93, 1A98, 1AA7, 1AAL, 1AAP, 1AAR, 1AAZ, 1AB8, 1ABR, 1AC6, 1ACB, 1AD1, 1AD3, 1AD4, 1ADB, 1ADC, 1ADE, 1ADI, 1ADU, 1ADV, 1AE1, 1AFW, 1AH8, 1AHE, 1AHH, 1AHW, 1AJ8, 1ALL, 1ALV, 1AMH, 1AOF, 1AOG, 1AOH, 1AOJ, 1AQ6, 1AQU, 1AR0, 1AT3, 1ATN, 1AUI, 1AUO, 1AUS, 1AV5, 1AVB, 1AVW, 1AVZ, 1AXI, 1AY7, 1AZT, 1AZV, 1B00, 1B0P, 1B34, 1B3D, 1B49, 1B4K, 1B55, 1B57, 1B67, 1B6T, 1B78, 1B7G, 1B8A, 1B8G, 1B9M, 1BBH, 1BD0, 1BEB, 1BFT, 1BHJ, 1BHT, 1BIQ, 1BIS, 1BJF, 1BJM, 1BJN, 1BJW, 1BKJ, 1BKP, 1BLX, 1BM7, 1BMQ, 1BNC, 1BND, 1BQL, 1BRC, 1BRL, 1BRS, 1BSL, 1BTH, 1BU7, 1BUC, 1BVK, 1BVN, 1BW0, 1BXG, 1BYF, 1BYK, 1C0F, 1C14, 1C1Y, 1C39, 1C3A, 1C3B, 1C3C, 1C8U, 1C94, 1CBK, 1CDT, 1CGI, 1CHO, 1CI1, 1CI4, 1CL1, 1CL5, 1CM5, 1CM7, 1CM9, 1CMB, 1CMV, 1CNQ, 1COL, 1COZ, 1CP2, 1CP9, 1CPJ, 1CQK, 1CQX, 1CSE, 1CXZ,

1D3Y, 1D6J, 1D6R, 1D8L, 1D9C, 1DAP, 1DBQ, 1DC3, 1DDR, 1DEK, 1DFJ, 1DGL, 1DHK, 1DJ0, 1DJN, 1DJO, 1DJS, 1DKF, 1DKL, 1DKR, 1DLM, 1DO6, 1DOK, 1DOR, 1DP4, 1DPG, 1DPM, 1DQJ, 1DQN, 1DQR, 1DQS, 1DS6, 1DTD, 1DXE, 1DZR, 1E0B, 1E2I, 1E7N, 1E8I, 1EBF, 1EBG, 1ECC, 1EDH, 1EEJ, 1EFU, 1EFV, 1EG5, 1EG9, 1EHI, 1EHW, 1EI1, 1EK1, 1EK6, 1ELQ, 1EN7, 1EO6, 1EO8, 1EQ9, 1ERN, 1ETK, 1ETT, 1EUV, 1EV7, 1EVK, 1EVX, 1EXT, 1EYV, 1EYZ, 1EZQ, 1F0Y, 1F2T, 1F37, 1F39, 1F3A, 1F3V, 1F4O, 1F5M, 1F5W, 1F60, 1F61, 1F6Y, 1F8X, 1F9Z, 1FBI, 1FBT, 1FBY, 1FDH, 1FGX, 1FIC, 1FIN, 1FLE, 1FLM, 1FO4, 1FQ1, 1FSS, 1FTL, 1FUP, 1G6N, 1GAE, 1GAR, 1GBI, 1GDH, 1GFL, 1GHA, 1GI9, 1GLA, 1GNW, 1GOT, 1GPE, 1GQK, 1GRN, 1GSD, 1HBI, 1HDM, 1HGX, 1HUL, 1HXP, 1IAI, 1IAR, 1IGC, 1ING, 1IPW, 1ITB, 1IVY, 1JHL, 1JKM, 1KBA, 1KKL, 1KNY, 1KSI, 1KWA, 1KXQ, 1KXT, 1KXV, 1L0Y, 1LBE, 1LCP, 1LLD, 1LPB, 1MAH, 1MAS, 1MEL, 1MJH, 1MKA, 1MLC, 1MSB, 1MVP, 1MYK, 1NCA, 1NMB, 1NSY, 1OME, 1ORO, 1PCZ, 1PD2, 1PDK, 1PFK, 1PHN, 1POW, 1PPE, 1PPF, 1PRG, 1PRX, 1PSR, 1PVD, 1PYM, 1QAV, 1QFU, 1QGK, 1QS0, 1R2F, 1RVE, 1SCJ, 1SES, 1SLU, 1SMP, 1SMT, 1SND, 1SPB, 1SPP, 1STF, 1TAB, 1TAF, 1TCR, 1TGS, 1TMK, 1TMQ, 1TPL, 1TVD, 1UDI, 1UGH, 1VHI, 1WEJ, 1WHS, 1WQ1, 1WWA, 1XCA, 1YAC, 1YCS, 1YFO, 1YHA, 2AAC, 2APS, 2BTF, 2GSA, 2GVB, 2IAD, 2JEL, 2KAI, 2KIN, 2MTA, 2PCC, 2POL, 2PTC, 2SIC, 2SNI, 2SPC, 2SQC, 2TEC, 2UGI, 2UTG, 2VIR, 2VIU, 3HHR, 3LAD, 3LYN, 4HTC, 4MDH, 4SGB, 4TSU, 6CSC, 6INS, 8CAT, 9ATC

Heterodimers list:

15C8, 1A0O, 1A10, 1A22, 1A2X, 1A3L, 1A4F, 1A50, 1A5F, 1A6D, 1A6E, 1A6U, 1A6W, 1A7N, 1A7O, 1A7P, 1A7Q, 1A7R, 1A93, 1ABR, 1ACB, 1AHW, 1ALL, 1ATN, 1AUI, 1AUS, 1AVW, 1AVZ, 1AXI, 1AY7, 1B34, 1BLX, 1BMQ, 1BND, 1BQL, 1BRC, 1BRL, 1BRS, 1BTH, 1BVK, 1BVN, 1C0F, 1C1Y, 1C3A, 1CGI, 1CHO, 1CP9, 1CSE, 1CXZ, 1D6R, 1DFJ, 1DHK, 1DJS, 1DKF, 1DQJ, 1DS6, 1DTD, 1EFU, 1EFV, 1EG9, 1EO8, 1ETT, 1EUV, 1EZQ, 1F2T, 1F3V, 1F60, 1FBI, 1FDH, 1FIN, 1FLE, 1FQ1, 1FSS, 1GBI, 1GHA, 1GI9, 1GLA, 1GOT, 1GRN, 1HDM, 1IAI, 1IAR, 1IGC, 1ITB, 1JHL, 1KKL, 1KXQ, 1KXT, 1KXV, 1L0Y, 1LPB, 1MAH, 1MEL, 1MLC, 1NCA, 1NMB, 1PDK, 1PHN, 1PPE, 1PPF, 1QAV, 1QFU, 1QGK, 1QS0, 1SCJ, 1SLU, 1SMP, 1SPB, 1SPP, 1STF, 1TAB, 1TAF, 1TCR, 1TGS, 1TMQ, 1UDI, 1UGH, 1WEJ, 1WHS, 1WQ1, 1YCS, 2BTF, 2IAD, 2JEL, 2KAI, 2KIN, 2MTA, 2PCC, 2PTC, 2SIC, 2SNI, 2TEC, 2VIR, 2VIU, 3HHR, 4HTC, 4MDH, 4SGB, 9ATC

Heterodimer monomers belonging to dimers with high sequence length ratios (ratio > 2):

1A10_E, 1A2X_A, 1ACB_E, 1AHW_D, 1AHW_F, 1AUI_A, 1AUI_B, 1AUS_L, 1AUS_S, 1BQL_L, 1BQL_Y, 1BRC_E, 1BTH_L, 1BVN_P, 1C0F_A, 1C0F_S, 1C1Y_A, 1CGI_E, 1CHO_E, 1CP9_A, 1CP9_B, 1CSE_E, 1CXZ_A, 1CXZ_B, 1D6R_A, 1DFJ_E, 1DFJ_I, 1DHK_A, 1DHK_B, 1DQJ_A, 1DQJ_C, 1DTD_A, 1EG9_A, 1EG9_B, 1ETT_H, 1EUV_A, 1EZQ_A, 1F60_A, 1F60_B, 1FBI_L, 1FBI_X, 1FLE_E, 1FSS_A, 1GBI_A, 1GHA_E, 1GI9_B, 1GLA_F, 1GLA_G, 1IGC_L, 1ITB_A, 1ITB_B, 1KKL_A, 1KKL_H, 1KXQ_D, 1KXQ_E, 1KXT_A, 1KXT_B, 1KXV_A, 1KXV_C, 1LPB_A, 1LPB_B, 1MAH_A, 1MLC_A, 1MLC_E, 1PPE_E, 1PPF_E, 1QGK_A, 1SCJ_A, 1SMP_A, 1SMP_I, 1SPB_S, 1STF_E, 1STF_I, 1TAB_E, 1TGS_Z, 1TMQ_A, 1TMQ_B, 1UDI_E, 1UDI_I, 1UGH_E, 1UGH_I, 1WEJ_F, 1WEJ_L, 2BTF_A, 2BTF_P, 2JEL_L, 2JEL_P, 2KAI_A, 2KIN_A, 2KIN_B, 2MTA_A, 2MTA_H, 2PCC_A, 2PCC_B, 2PTC_E, 2SIC_E, 2SIC_I, 2SNI_E, 2TEC_E, 4HTC_L, 4SGB_E, 9ATC_A, 9ATC_B

Supplementary material tables

a	b	c	d	e	f	g	h
1	1QGK_A	876	28	88.58	36.83	25.00	49.77
2	1BVN_P	496	10	62.75	45.94	20.56	49.40
3	1SMP_A	468	8	53.06	46.22	20.94	47.65
4	1DFJ_I	456	8	67.42	37.65	28.95	46.27
5	1EG9_A	447	11	54.78	40.96	25.73	44.52
6	1F60_A	440	7	52.50	49.06	27.27	50.00
7	1AUS_L	439	9	52.46	45.11	27.79	47.15
8	1WEJ_L	437	7	92.73	42.67	12.59	48.97
9	1IGC_L	435	10	54.35	46.53	10.57	47.36
10	1FBI_L	435	7	58.21	43.75	15.40	45.98
11	1MLC_A	432	6	68.85	42.05	14.12	45.83
12	1AHW_D	428	6	66.15	39.12	15.19	43.22
13	1BQL_L	426	8	68.42	46.34	13.38	49.30
14	2JEL_L	425	5	60.00	41.89	12.94	44.24
15	1DQJ_A	424	7	64.52	43.37	14.62	46.46
16	2BTF_A	374	9	51.95	38.38	20.59	41.18
17	1C0F_A	367	9	62.82	45.33	21.25	49.05
18	1KKL_A	335	7	50.77	37.78	19.40	40.30
19	9ATC_A	310	4	61.70	45.25	15.16	47.74
20	1DTD_A	303	5	71.08	30.45	27.39	41.58
21	1SCJ_A	275	6	68.75	48.60	34.91	55.64
22	2SNI_E	275	9	76.92	49.46	33.09	58.55
23	2SIC_E	275	8	77.17	43.17	33.45	54.55
24	1A10_E	274	7	79.07	44.68	31.39	55.47
25	1CSE_E	274	8	78.72	43.33	34.31	55.47
26	1CGI_E	245	5	63.64	49.68	35.92	54.69
27	1ACB_E	241	9	72.62	44.59	34.85	54.36
28	1BTH_L	240	7	71.91	49.67	37.08	57.92
29	1CHO_E	238	7	68.24	40.52	35.71	50.42
30	1GHA_E	236	7	81.67	50.00	25.42	58.05
31	1ETT_H	231	5	53.33	39.18	25.97	42.86
32	1FLE_E	229	5	60.24	30.14	36.24	41.05
33	2KAI_A	223	5	71.08	32.14	37.22	46.64
34	1UGH_E	223	8	78.57	37.91	31.39	50.67
35	1TGS_Z	222	6	59.34	39.69	40.99	47.75
36	1D6R_A	220	5	58.33	38.97	38.18	46.36
37	1PPE_E	220	7	65.91	35.61	40.00	47.73
38	1TAB_E	220	6	55.68	31.82	40.00	41.36
39	1BRC_E	220	6	75.29	47.41	38.64	58.18

40	2PTC_E	220	5	57.32	31.16	37.27	40.91
41	1PPF_E	212	6	76.32	48.53	35.85	58.49
42	1STF_E	212	8	73.75	43.18	37.74	54.72
43	1DHK_B	195	4	71.43	14.43	50.26	43.08
44	1GBI_A	170	3	57.69	33.90	30.59	41.18
45	4SGB_E	168	5	76.06	35.05	42.26	52.38
46	1ITB_A	153	4	52.48	48.08	66.01	50.98
47	2BTF_P	139	4	67.69	28.38	46.76	46.76
48	1MLC_E	129	4	83.33	32.18	32.56	48.84
49	1BQL_Y	129	4	76.19	41.38	32.56	52.71
50	1C0F_S	127	6	67.61	48.21	55.91	59.06
51	1KXV_C	119	1	51.85	46.15	45.38	48.74
52	1TMQ_B	117	2	57.89	43.33	48.72	50.43
53	1KXT_B	109	3	67.27	50.00	50.46	58.72
54	2PCC_B	108	5	55.10	33.90	45.37	43.52
55	2MTA_A	105	3	53.33	40.00	42.86	45.71
56	1WEJ_F	104	6	64.10	43.08	37.50	50.96
57	1SMP_I	100	3	55.56	29.09	45.00	41.00
58	1STF_I	95	5	71.79	48.21	41.05	57.89
59	1F60_B	90	3	64.56	9.09	87.78	57.78
60	1CXZ_B	86	1	80.39	37.14	59.30	62.79
61	1KKL_H	86	2	65.31	40.54	56.98	54.65
62	2JEL_P	85	3	86.11	28.57	42.35	52.94
63	1LPB_A	85	2	53.19	44.74	55.29	49.41
64	1UDI_I	83	2	60.32	10.00	75.90	48.19
65	1UGH_I	82	1	77.05	23.81	74.39	63.41

Table S1. The summary of good predictions. The columns are as follows:

a) Index,
b) PDB id code,
c) Chain length,
d) Number of fast modes,
e) Percent of true predictions,
f) Percent of false predictions,
g) Percent of targets out of all residues in the chain,
h) Percent of predictions out of all residues in the chain.

							Adjustable GNM					Statistical potential				Combination			
**a**	**b**	**c**	**d**	**e**	**f**	**g**	**h**	**i**	**j**	**k**	**l**	**m**	**n**	**o**	**p**	**q**	**r**	**s**	**t**
		len. 1	len. 2	nea	Best r	Cov.	Cov %	Best	Cov.	Cov %	Best	Cov.	Cov %	Best	Cov.	Cov %	Best r	Cov	Cov %
1	1avw_A_B	220	172	10	10	1	10%	90	0	0%	1	8	80%	1	5	50%	64	0	0%
2	1bui_A_C	247	121	10	9	2	20%	31	0	0%	9	1	10%	2	3	30%	16	0	0%
3	1bui_B_C	247	121	10	34	0	0%	46	0	0%	10	1	10%	21	0	0%	13	0	0%
4	1bvn_P_T	495	74	10	60	0	0%	1	2	20%	2	8	80%	16	0	0%	1	8	80%
5	1cho_E_I	236	56	10	2	6	60%	6	3	30%	3	5	50%	1	7	70%	1	5	50%
6	1dfj_I_E	456	123	9	26	0	0%	67	0	0%	1	6	67%	2	6	67%	31	0	0%
7	1e96_B_A	192	181	10	65	0	0%	26	0	0%	3	4	40%	39	0	0%	4	2	20%
8	1ewy_A_C	295	98	10	6	3	30%	23	0	0%	7	4	40%	1	8	80%	7	4	40%
9	1f6m_A_C	316	108	10	71	0	0%	25	0	0%	44	0	0%	60	0	0%	47	0	0%
10	1fm9_D_A	272	212	10	9	2	20%	40	0	0%	1	6	60%	3	4	40%	9	1	10%
11	1g6v_A_K	259	126	6	98	0	0%	94	0	0%	31	0	0%	90	0	0%	72	0	0%
12	1gpq_D_A	129	128	10	18	0	0%	2	1	10%	33	0	0%	12	0	0%	13	0	0%
13	1gpw_A_B	253	200	10	53	0	0%	11	0	0%	3	4	40%	17	0	0%	2	5	50%
14	1he1_C_A	181	131	10	45	0	0%	10	1	10%	5	1	10%	17	0	0%	2	4	40%
15	1he8_A_B	841	166	1	14	0	0%	100	0	0%	21	0	0%	4	0	0%	81	0	0%
16	1ku6_A_B	535	61	10	26	0	0%	91	0	0%	1	7	70%	2	6	60%	56	0	0%
17	1ma9_A_B	455	360	10	26	0	0%	4	5	50%	1	8	80%	1	5	50%	1	7	70%
18	1nbf_A_D	323	70	10	73	0	0%	42	0	0%	15	0	0%	39	0	0%	11	0	0%
19	1oph_A_B	372	220	10	3	5	50%	1	5	50%	1	9	90%	1	5	50%	1	10	100%
20	1ppf_E_I	210	56	10	1	10	100%	52	0	0%	7	1	10%	1	6	60%	6	2	20%
21	1r0r_E_I	274	51	10	14	0	0%	21	0	0%	2	7	70%	1	7	70%	5	1	10%
22	1s6v_A_B	291	108	4	10	0	0%	10	0	0%	2	1	25%	4	1	25%	2	2	50%
23	1t6g_A_C	362	182	10	40	0	0%	33	0	0%	7	1	10%	10	1	10%	32	0	0%
24	1tmq_A_B	470	117	10	48	0	0%	97	0	0%	1	6	60%	14	0	0%	65	0	0%
25	1tx6_A_I	220	120	10	55	0	0%	83	0	0%	29	0	0%	36	0	0%	71	0	0%
26	1u7f_B_A	190	178	10	42	0	0%	41	0	0%	14	0	0%	26	0	0%	18	0	0%
27	1ugh_E_I	223	83	10	9	2	20%	5	1	10%	1	6	60%	1	6	60%	1	4	40%
28	1w1i_A_F	728	349	4	58	0	0%	15	0	0%	4	1	25%	28	0	0%	1	4	100%
29	1wq1_G_R	324	166	10	4	2	20%	68	0	0%	4	2	20%	6	3	30%	36	0	0%
30	1xd3_A_B	206	70	10	36	0	0%	74	0	0%	1	10	100%	7	3	30%	47	0	0%
31	1yvb_A_I	241	108	10	9	1	10%	14	0	0%	1	9	90%	1	6	60%	1	8	80%
32	2a5t_A_B	281	278	1	101	0	0%	96	0	0%	11	0	0%	86	0	0%	73	0	0%
33	2bkr_A_B	210	74	10	86	0	0%	6	1	10%	3	1	10%	52	0	0%	1	7	70%
34	2btf_A_P	364	139	10	27	0	0%	5	2	20%	2	7	70%	9	2	20%	1	7	70%
35	2ckh_A_B	225	72	10	57	0	0%	44	0	0%	7	3	30%	17	0	0%	7	1	10%
36	2fi4_E_I	220	58	10	5	1	10%	100	0	0%	6	2	20%	2	7	70%	66	0	0%
37	2goo_A_C	103	92	10	100	0	0%	34	0	0%	13	0	0%	86	0	0%	31	0	0%
38	2sni_E_I	275	65	10	3	6	60%	19	0	0%	2	6	60%	1	10	100%	1	2	20%
39	3fap_A_B	107	92	10	54	0	0%	27	0	0%	5	2	20%	27	0	0%	16	0	0%
40	3pro_A_C	170	142	10	12	0	0%	38	0	0%	12	0	0%	12	0	0%	22	0	0%
41	3sic_E_I	275	108	10	2	5	50%	55	0	0%	1	8	80%	1	10	100%	21	0	0%
		Averages			34.7	1.1	11.2%	40.2	0.5	5.1%	8.0	3.5	36.3%	18.5	2.7	27.6%	23.3	2.0	22.7%

Table S2. The efficiency of the adjustable prediction algorithm (3D algorithm with variable number of modes) with the Vakser decoy sets. The columns are as follows:

a) Decoy set number,

b) Decoy set name (pdb ID code followed by two chain letters),

c) Longer chain’s length,

d) Shorter chain’s length,

e) The number of near native structures in a decoy set,

f) (3D adjustable algorithm) the best standing of the longer chain belonging to one of the near native decoys,

g) (3D adjustable algorithm) the coverage expressed as the number of correctly predicted longer chains belonging to near native decoys among the first *n* predictions, where *n* is the number of near native decoys (column e),

h) (3D adjustable algorithm) the coverage (column g), expressed as the percentage,

i) (3D adjustable algorithm) the best standing of the shorter chain belonging to one of the near native decoys,

j) (3D adjustable algorithm) the coverage expressed as the number of correctly predicted shorter chains belonging to near native decoys among the first *n* predictions, where *n* is the number of near native decoys (column e),

k) (3D adjustable algorithm) the coverage (column i), expressed as the percentage,

l) (Statistical potential) the best standing of the longer chain belonging to one of the near native decoys,

m) (Statistical potential) the coverage expressed as the number of correctly predicted longer chains belonging to near native decoys among the first *n* predictions, where *n* is the number of near native decoys (column e),

n) (Statistical potential) the coverage (column m), expressed as the percentage,

o) (3D approach combined with Statistical potential) the best standing of the longer chain belonging to one of the near native decoys,

p) (3D approach combined with Statistical potential) the coverage expressed as the number of correctly predicted longer chains belonging to near native decoys among the first *n* predictions, where *n* is the number of near native decoys (column e),

q) (3D approach combined with Statistical potential) the coverage (column p), expressed as the percentage.

							Adjustable GNM				Stat. potential			Combination					
**a**	**b**	**c**	**d**	**e**	**f**	**g**	**h**	**i**	**j**	**k**	**l**	**m**	**n**	**o**	**p**	**q**	**r**	**s**	**t**
No.	Name	sz1	sz2	nn	nb1	Cov	Cov	nb2	Cov	Cov	nb1	Cov	Cov	nb1	Cov	Cov	nb2	Cov	Cov
1	1AVZ	99	57	4	68	0	0.0%	72	0	0.0%	22	0	0.0%	42	0	0.0%	58	0	0.0%
2	1BGS	108	89	4	44	0	0.0%	83	0	0.0%	3	1	25.0%	13	0	0.0%	50	0	0.0%
3	1BRC	220	56	4	3	1	25.0%	88	0	0.0%	1	3	75.0%	1	3	75.0%	59	0	0.0%
4	1CGI	245	56	4	31	0	0.0%	69	0	0.0%	1	2	50.0%	19	0	0.0%	43	0	0.0%
5	1DFJ	456	124	4	49	0	0.0%	27	0	0.0%	2	1	25.0%	21	0	0.0%	8	0	0.0%
6	1FSS	532	61	4	25	0	0.0%	70	0	0.0%	6	0	0.0%	4	1	25.0%	47	0	0.0%
7	1UGH	223	82	4	1	1	25.0%	1	2	50.0%	1	1	25.0%	1	1	25.0%	1	2	50.0%
8	1WQ1	320	166	4	2	2	50.0%	24	0	0.0%	11	0	0.0%	14	0	0.0%	5	0	0.0%
9	2PCC	291	108	4	32	0	0.0%	57	0	0.0%	4	1	25.0%	7	0	0.0%	28	0	0.0%
10	2SIC	275	107	4	2	1	25.0%	75	0	0.0%	1	2	50.0%	1	3	75.0%	46	0	0.0%
					26	0.5	12.5%	57	0.2	5.0%	5.2	1.1	27.5%	12	0.8	20.0%	35	0.2	5.0%

Table S3. The efficiency of the adjustable prediction algorithm (3D algorithm with variable number of modes) with the Sternberg decoy sets. The columns are as follows:

a) Decoy set number,

b) Decoy set name (pdb ID code),

c) Longer chain's length,

d) Shorter chain's length,

e) The number of near native structures in a decoy set,

f) (3D adjustable algorithm) the best standing of the longer chain belonging to one of the near native decoys,

g) (3D adjustable algorithm) the coverage expressed as the number of correctly predicted longer chains belonging to near native decoys among the first *n* predictions, where *n* is the number of near native decoys (column e),

h) (3D adjustable algorithm) the coverage (column g), expressed as the percentage,

i) (3D adjustable algorithm) the best standing of the shorter chain belonging to one of the near native decoys,

j) (3D adjustable algorithm) the coverage expressed as the number of correctly predicted shorter chains belonging to near native decoys among the first *n* predictions, where *n* is the number of near native decoys (column e),

k) (3D adjustable algorithm) the coverage (column i), expressed as the percentage,

l) (Statistical potential) the best standing of the longer chain belonging to one of the near native decoys,

m) (Statistical potential) the coverage expressed as the number of correctly predicted longer chains belonging to near native decoys among the first *n* predictions, where *n* is the number of near native decoys (column e),

n) (Statistical potential) the coverage (column m), expressed as the percentage,

o) (3D approach combined with Statistical potential) the best standing of the longer chain belonging to one of the near native decoys,

p) (3D approach combined with Statistical potential) the coverage expressed as the number of correctly predicted longer chains belonging to near native decoys among the first *n* predictions, where *n* is the number of near native decoys (column e),

q) (3D approach combined with Statistical potential) the coverage (column p), expressed as the percentage.

Supplementary material figures

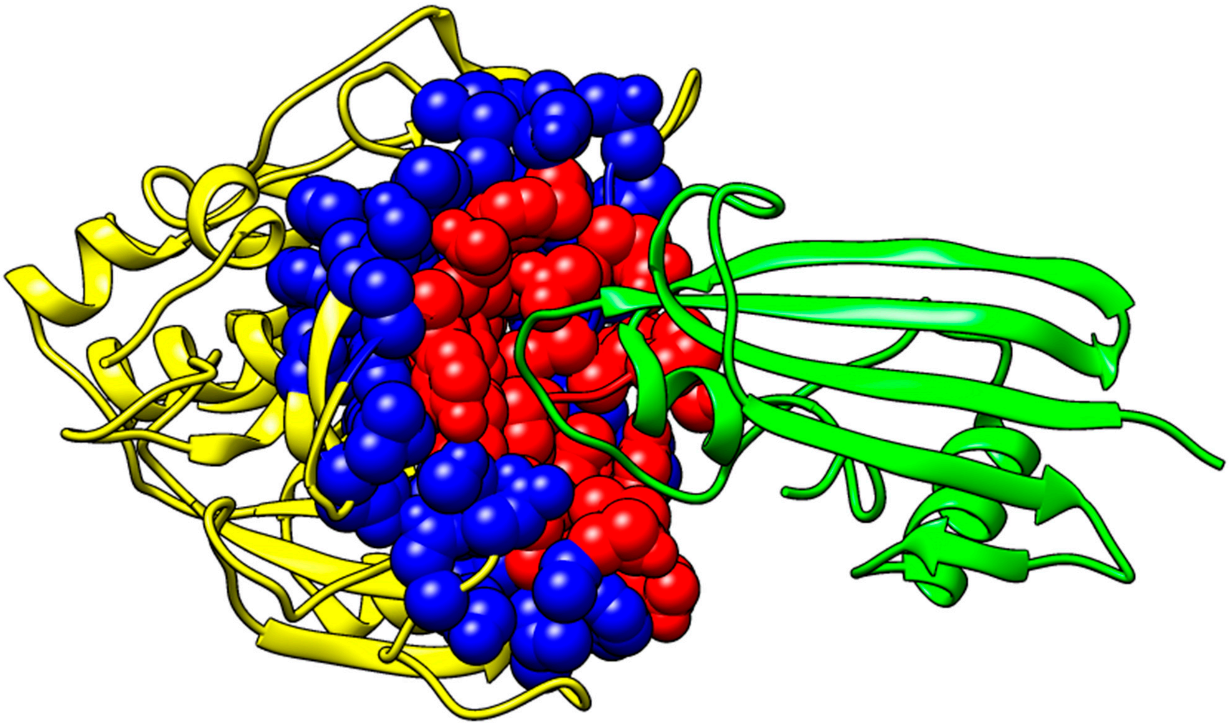

Figure S1. An illustration of the targets. The targets are depicted using the Subtilisin with its protein inhibitor Streptomyces from Bacillus amyloliquefaciens (pdb ID code 2SIC). There are two chains, I and E. The chain E is yellow and the chain I is green (both depicted as ribbons). The chain's E contact residues are colored red and visualized using the whole atom representation. Its first layer residues (residues in direct contact with the contact residues) are colored blue and visualized via the whole atom representation (van der Waals radii).

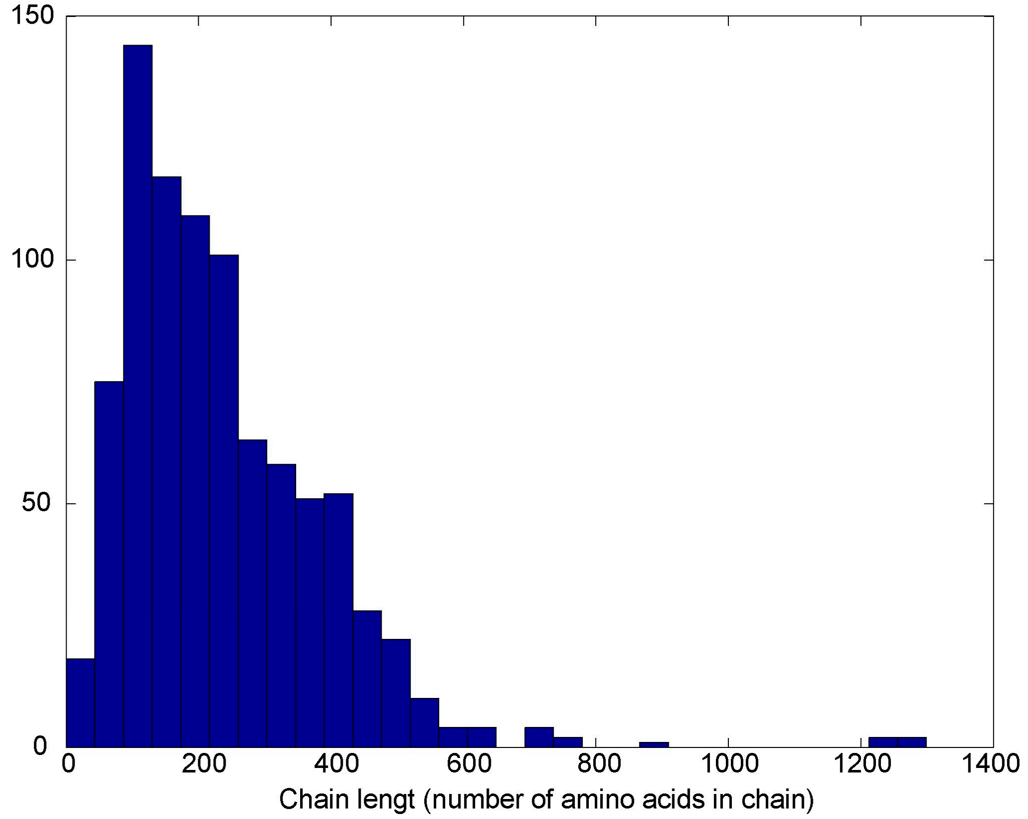

Figure S2. Protein chain lengths distribution for both heterodimers and homodimers.

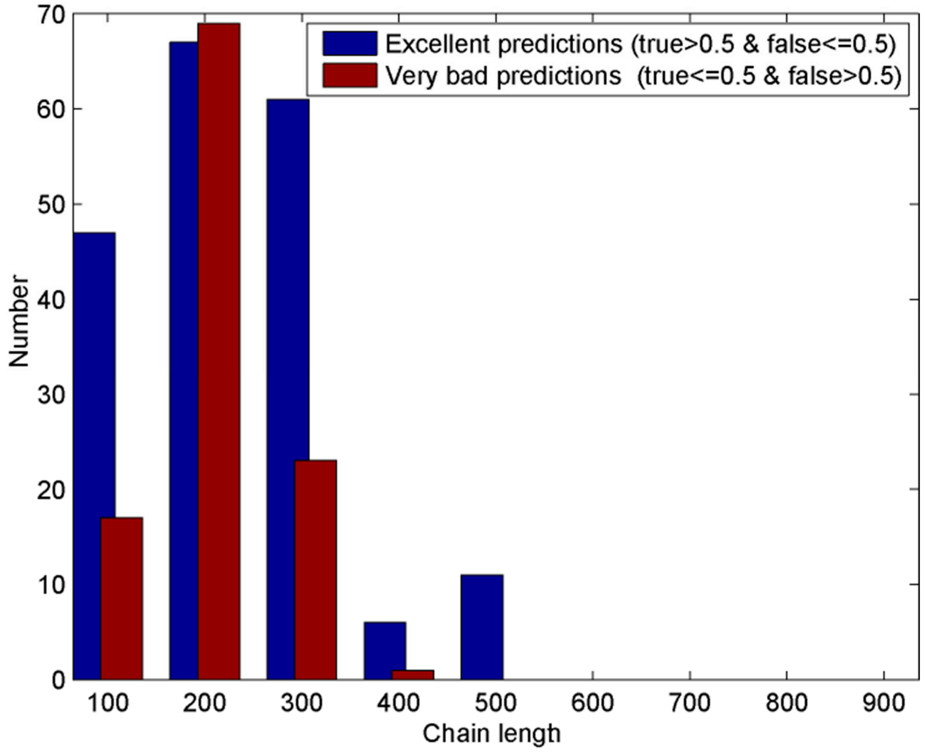

Figure S3. Prediction histogram based on the analysis of all chains over the sequence lengths for the simple prediction approach based on five fastest modes. Only good and very bad predictions are depicted. Blue bars are good predictions and red bars are very bad predictions. It is obvious that five modes do not offer good prediction because in some cases (chain longer that 100 and shorter than 200 amino acids) the number of bad predictions is higher than the number of good predictions.

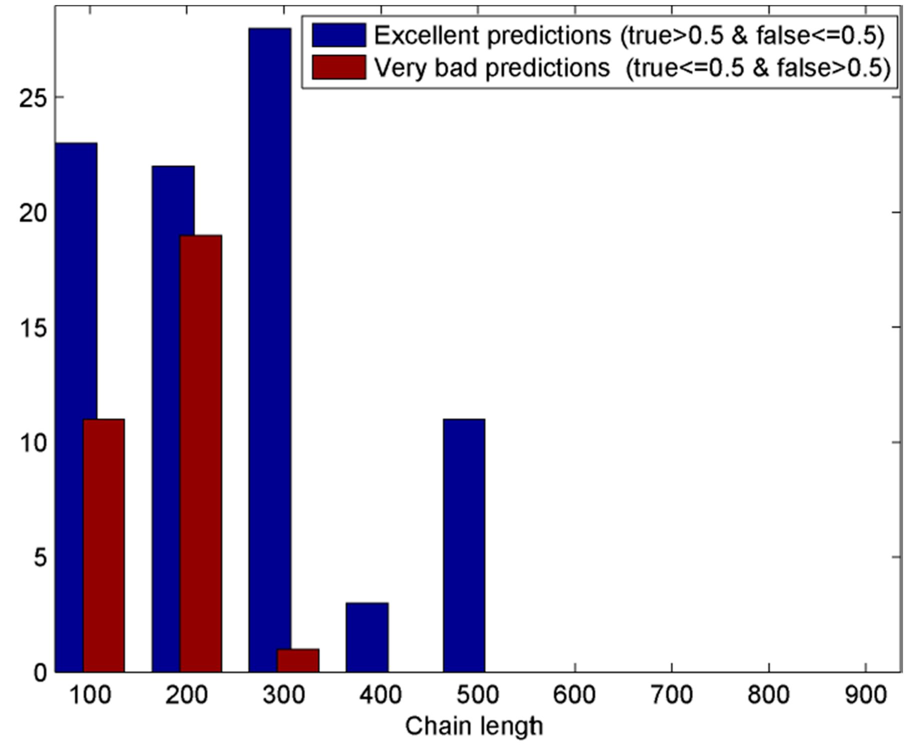

Figure S4. Prediction histogram for heterodimer chains only, for the simple prediction approach based on the 5 fastest modes. The prediction is better than with heterodimers and homodimers combined, but not satisfactory yet, because there is still less than 50 % of good predictions (31.29 % of good predictions – 87 chains and 11.15 % of bad ones – 31 chains).

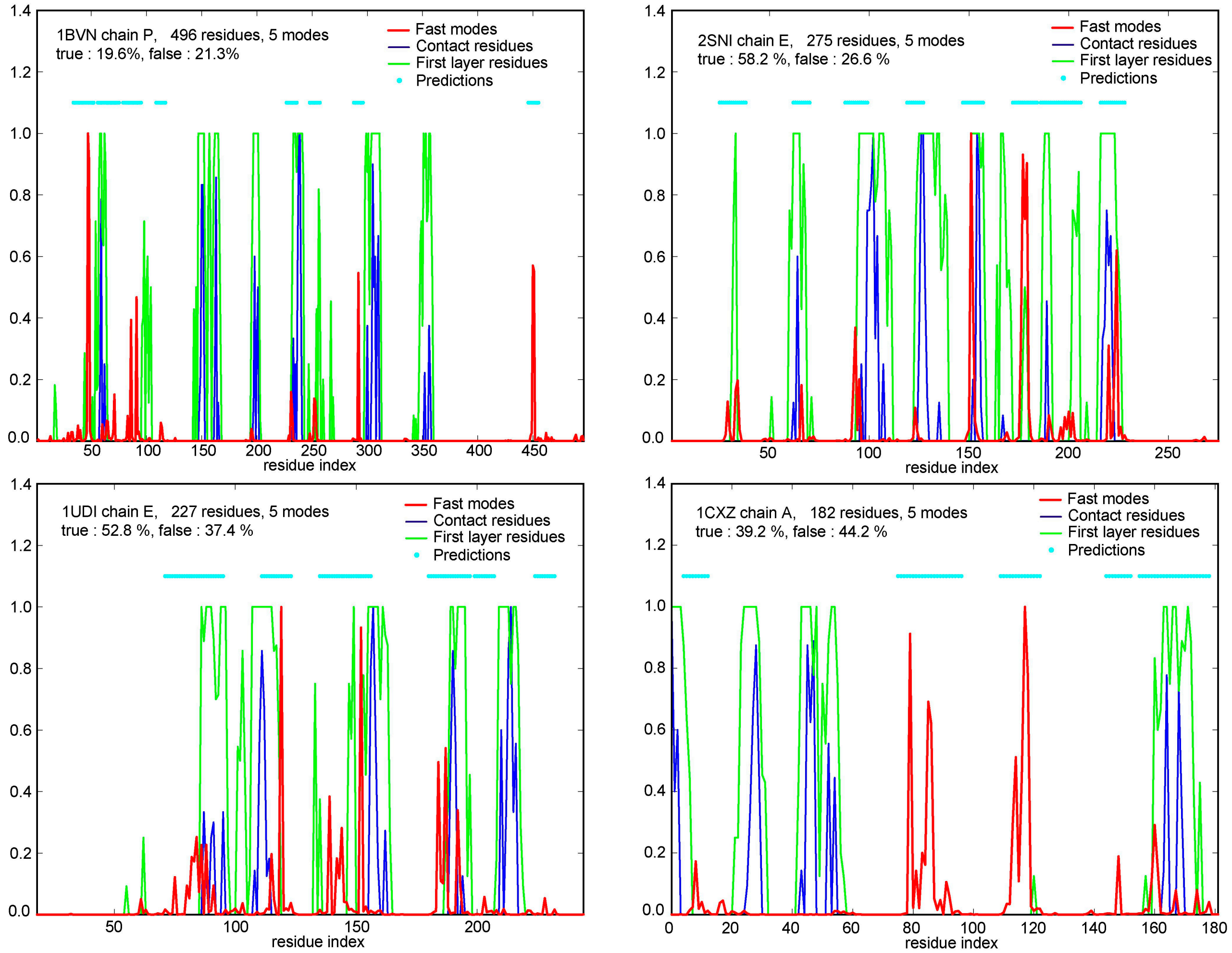

Figure S5. An example of the one dimensional, i.e., sequential approach to prediction, for 4 different chains (1BVN chain P, 2SNI chain E, 1UDI chain E and 1CXZ chain A). The kinetically hot residues are recognized via the weighted sum (Eq. 1) of fastest five modes per chain. Red lines depict the weighted sums. Blue lines are contacts residues. Green lines are first layer residues. Cyan dots are predictions. None of the chains have missing residues in the middle of their sequences.

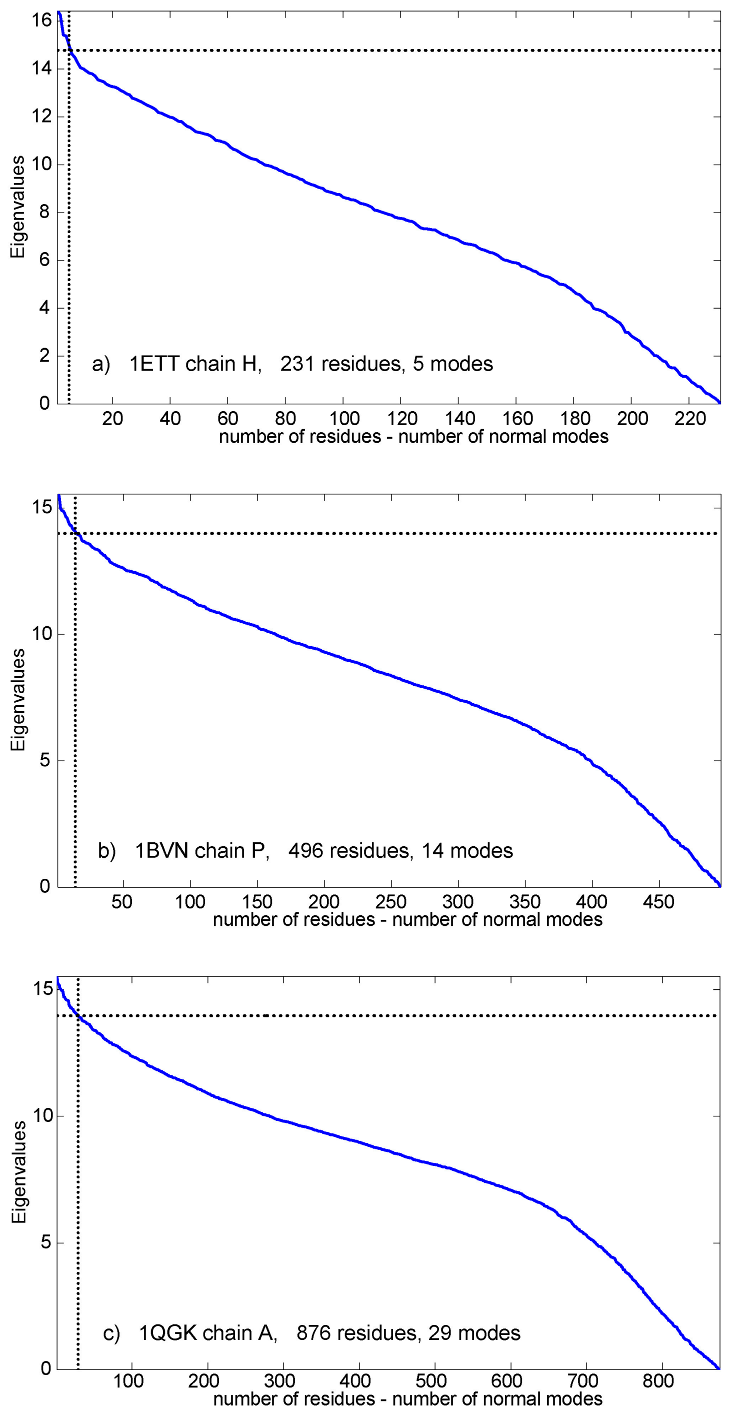

Figure S6. Distributions of eigenvalues for three different protein chains (dimer 1ETT chains H, dimer 1BVN chain P and dimer 1QGK chain A). The intersection of horizontal and vertical line on each plot designates the eigenvalues which cover top 10% of the eigenvalues span. It can be easily observed that top 10 % eigenvalues are covered by a different number of modes for each of these three chains. 5 modes correspond to top 10 % of eigenvalues only for 1ETT's chain H, 1BVN chain P requires 14 modes and chain A from dimer 1QGK requires 29 modes to cover 10% of modes.

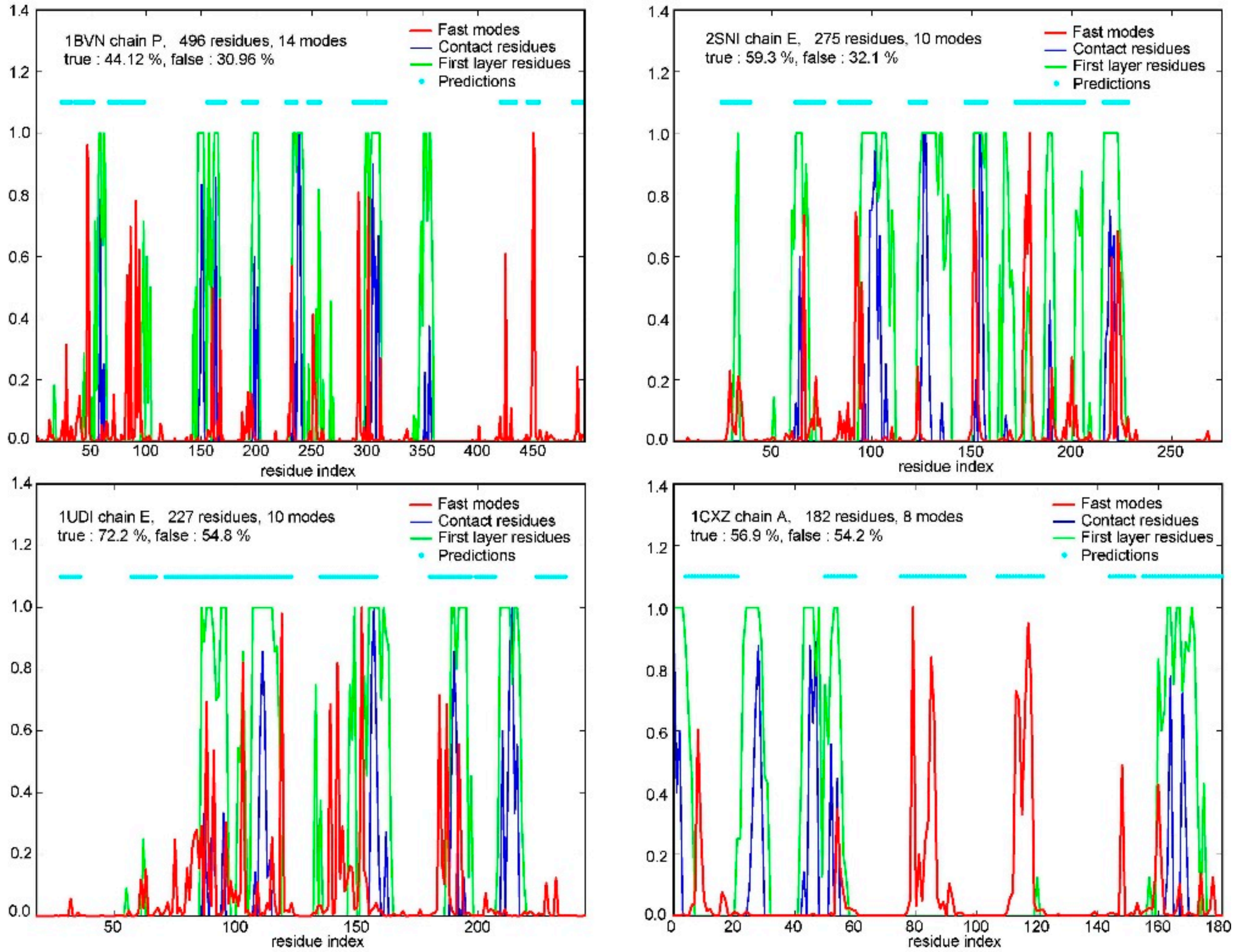

Figure S7. An example of the 1D prediction (sequential neighbors influence only) based on the fastest 10 % of modes per chain, for 4 different chains (1BVN chain P, 2SNI chain E, 1UDI chain E and 1CXZ chain A). Red lines depict the weighted sums. Blue lines are the contacts residues. Green lines depict the first layer residues. Cyan dots are the predictions.

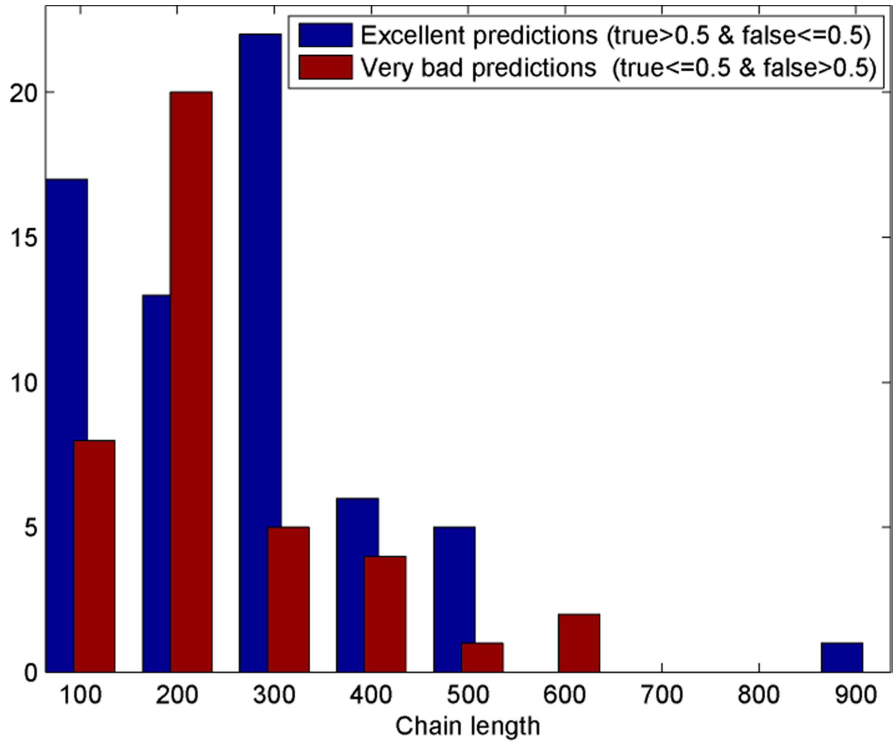

Figure S8. Prediction histogram for heterodimer chains only, for the prediction approach based on the modes that correspond to top 10 % of the eigenvalues span. There is still less than 50 % of good predictions and the distribution of predictions is slightly worse than the distribution for the five modes only (23.02 % of good predictions, 64 chains and 14.39 % of very bad predictions, 40 chains). However, a very long chain (1QGK – chain A, 876 residues) got into the category of good predictions.

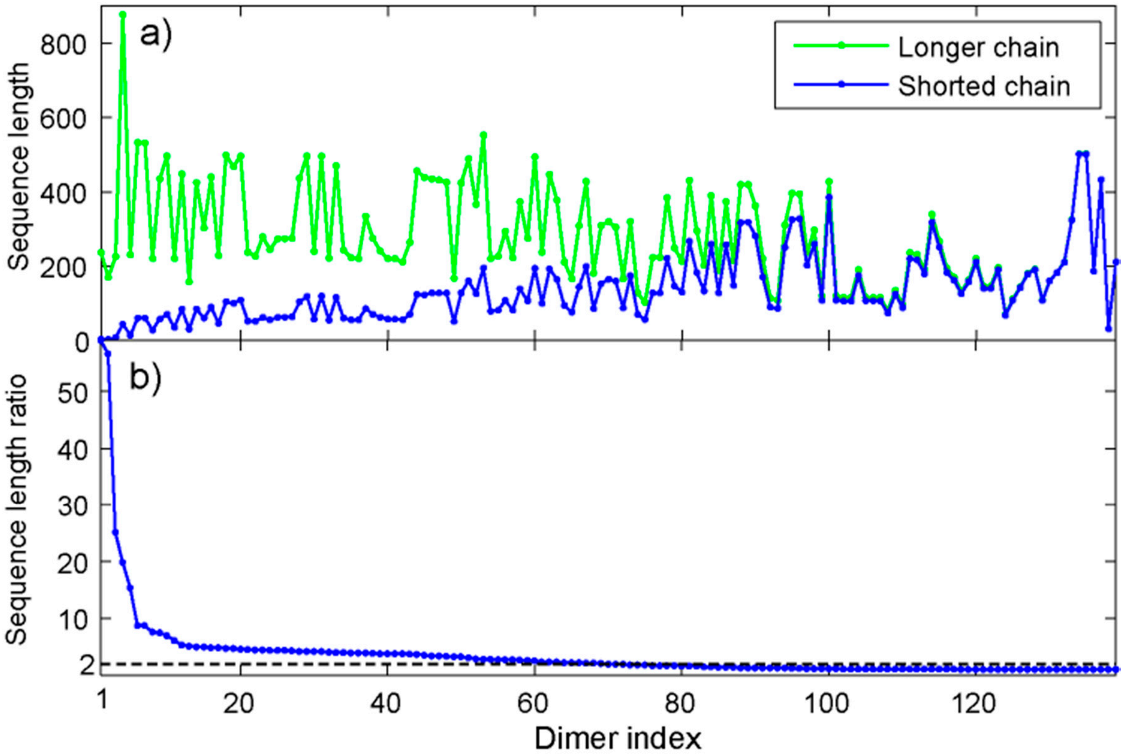

Figure S9. Dimer chain lengths for the set of 139 different heterodimers. a) Distribution of their chain lengths; longer chains are green, shorter chains are blue; b) and their corresponding sequence length ratios.

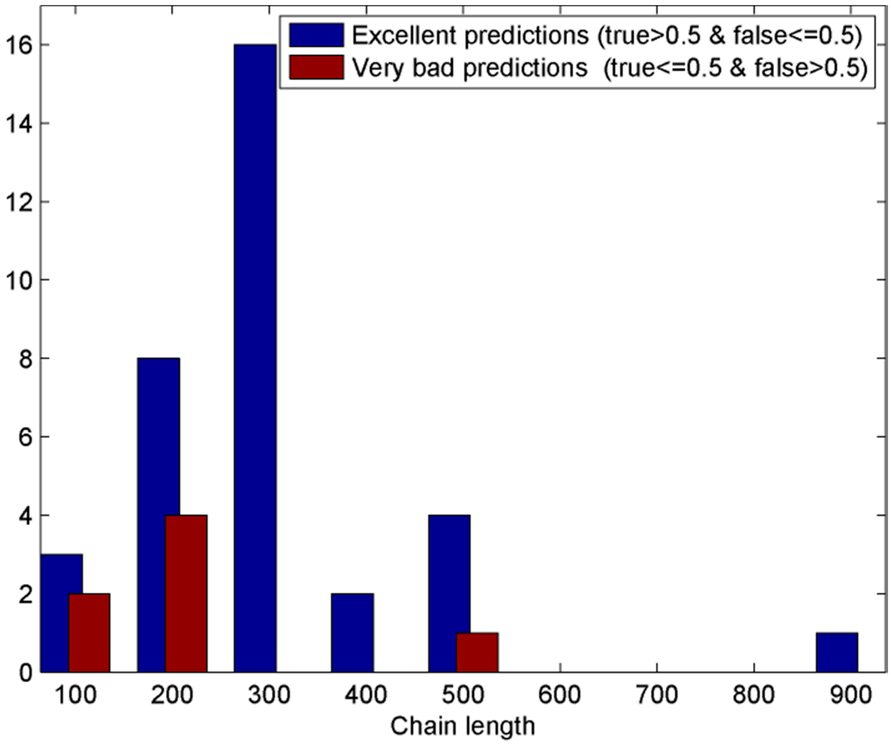

Figure S10. Prediction histogram, for chains in heterodimers with high sequence length ratios (length ratio > 2, chain length > 80 residues) for the prediction approach based on the modes which correspond to top 10 % of eigenvalues span. There is still less than 50 % of good predictions and the distribution is worse than the distribution for 5 modes only, 33.01 % of good predictions (34 out of 103 chains) and 6.8 % of very bad predictions (7 chains).

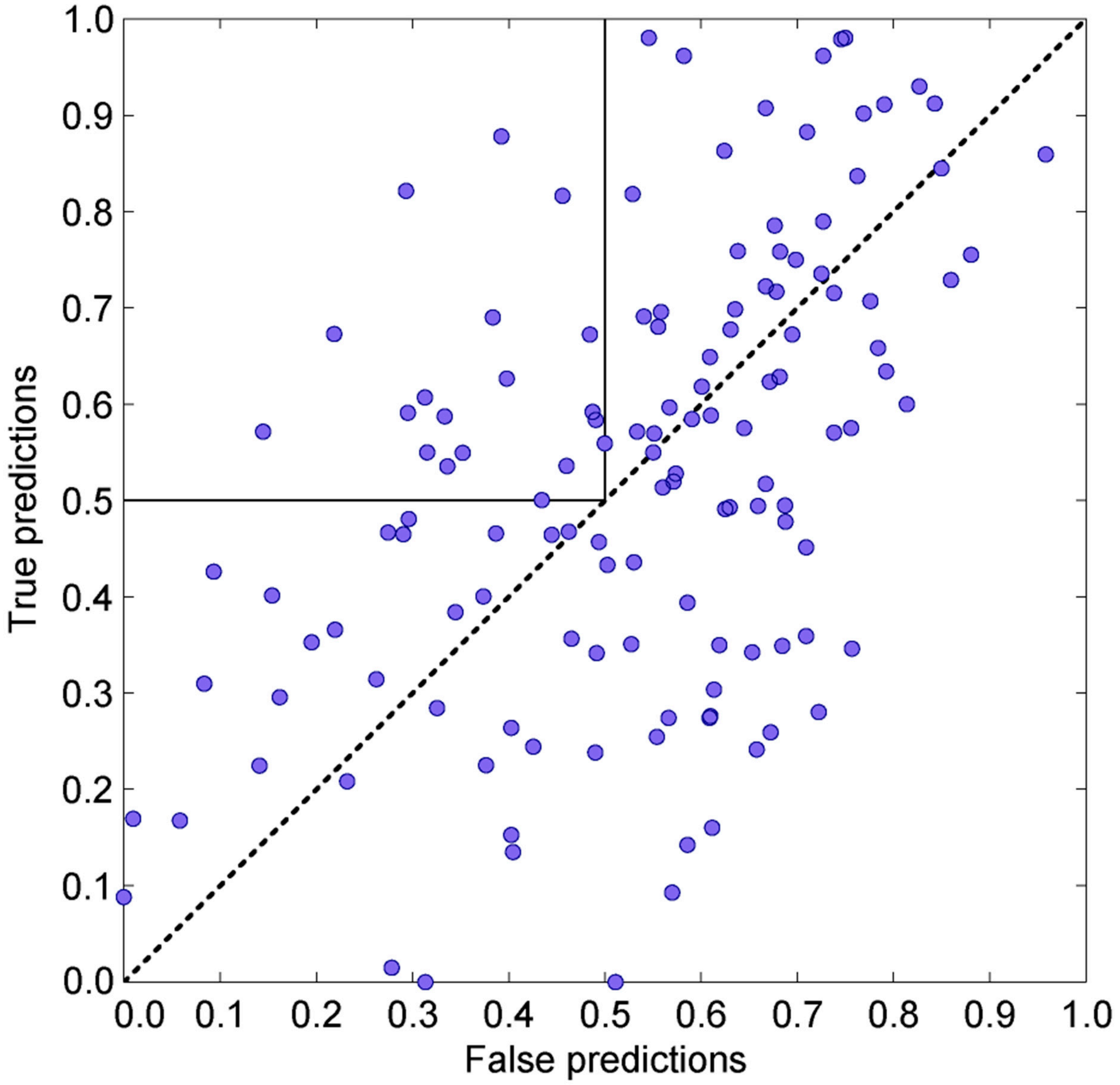

Figure S11. Prediction output for chains in heterodimers with low sequence length ratios (length ratio <= 2, chain length >80) for the prediction approach based on modes corresponding to top 10 % of eigenvalues range. The true positives mean is 52.75 %, and the false positives mean is 53.18 %. There is 13.64 % of good predictions (18 chains) and 20.45 % of very bad predictions (27 chains).

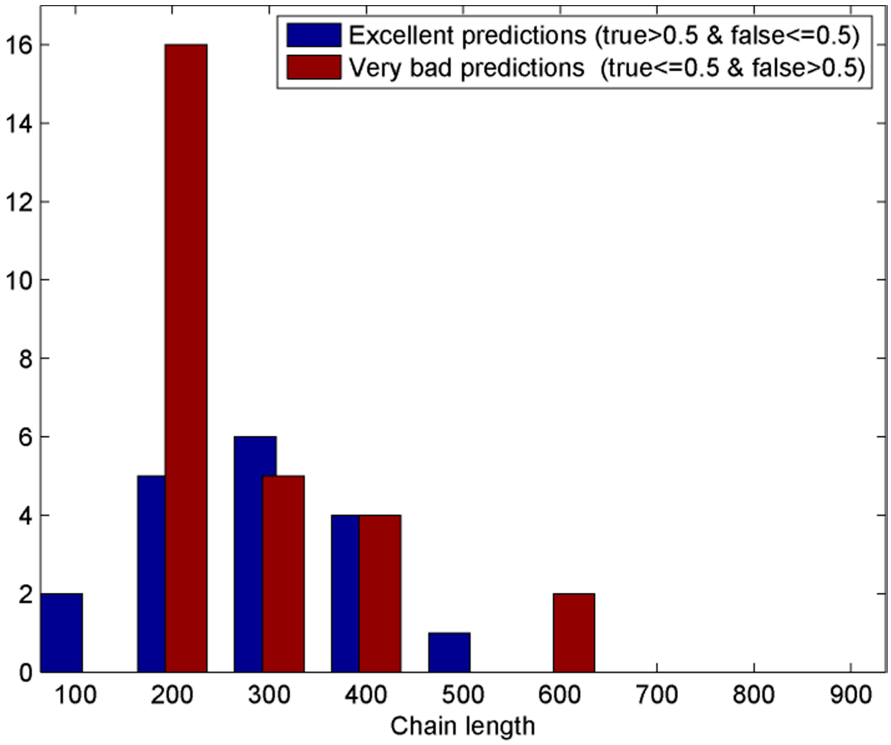

Figure S12. Prediction histogram over the sequence lengths for the simple prediction approach based on the fastest 10 % of modes for each chain, for chains in heterodimers with low sequence length ratios (length ratio <= 2, chain length >80). Blue bars are good predictions and red bars are very bad predictions. There is only 13.64 % of good predictions (18 chains of 132) to 20.45 % of very bad predictions (27 chains).

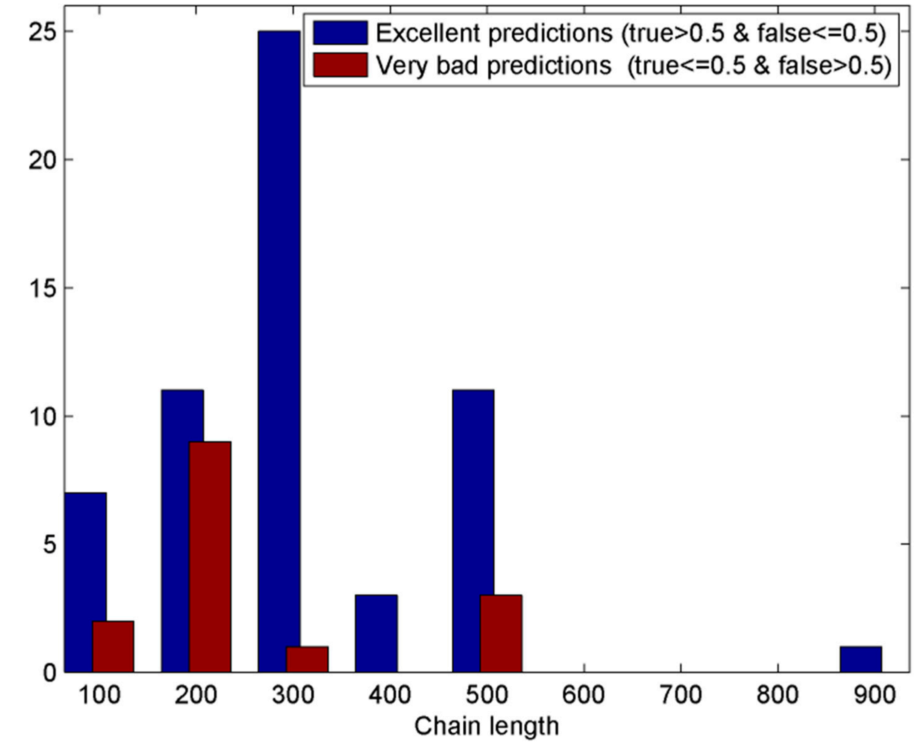

Figure S13. Histogram of predictions over the sequence lengths for the prediction approach based on the adjustable number of fast modes, with the 1D influence of hot residues, for chains in dimers with high sequence length ratio (length ratio > 2, length > 80 residues). The true positives mean true is 53.27 %, and false positives mean is 42.05 %. There is 56.31 % of good predictions (58 chains) and 14.56 % of very bad predictions (15 chains).

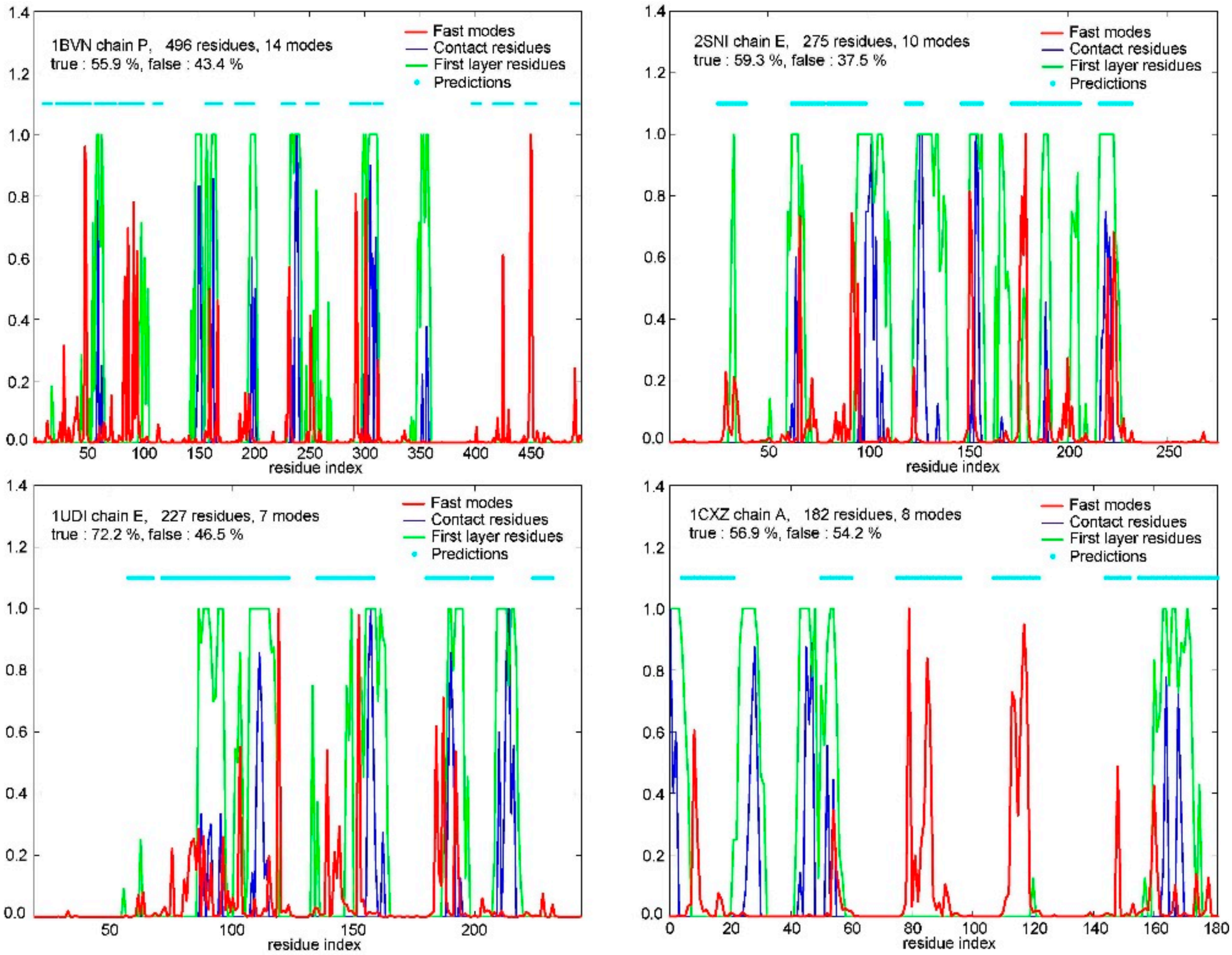

Figure S14. Examples of the prediction based on the adjustable number of fast modes and the sequential influence of hot residues. The four different chains are depicted (1BVN chain P, 2SNI chain E, 1UDI chain E and 1CXZ chain A). Red lines depict weighted sums. Blue lines designate contacts residues. Green lines are first layer residues. Cyan dots are predictions. For the three longest chains from that group, 1BVN chain P, 2SNI chain E, 1UDI chain E, the percent of true positives is over 50%, and percent of false positives is less than 50 % (the chain E of 1UDI, has a highest difference between true and false positives which is an indication of a high correlation between the kinetically hot residues and contact scaffolds for that chain). Only the shortest example, 1CXZ chain A, has both true and false positives over 50 %.

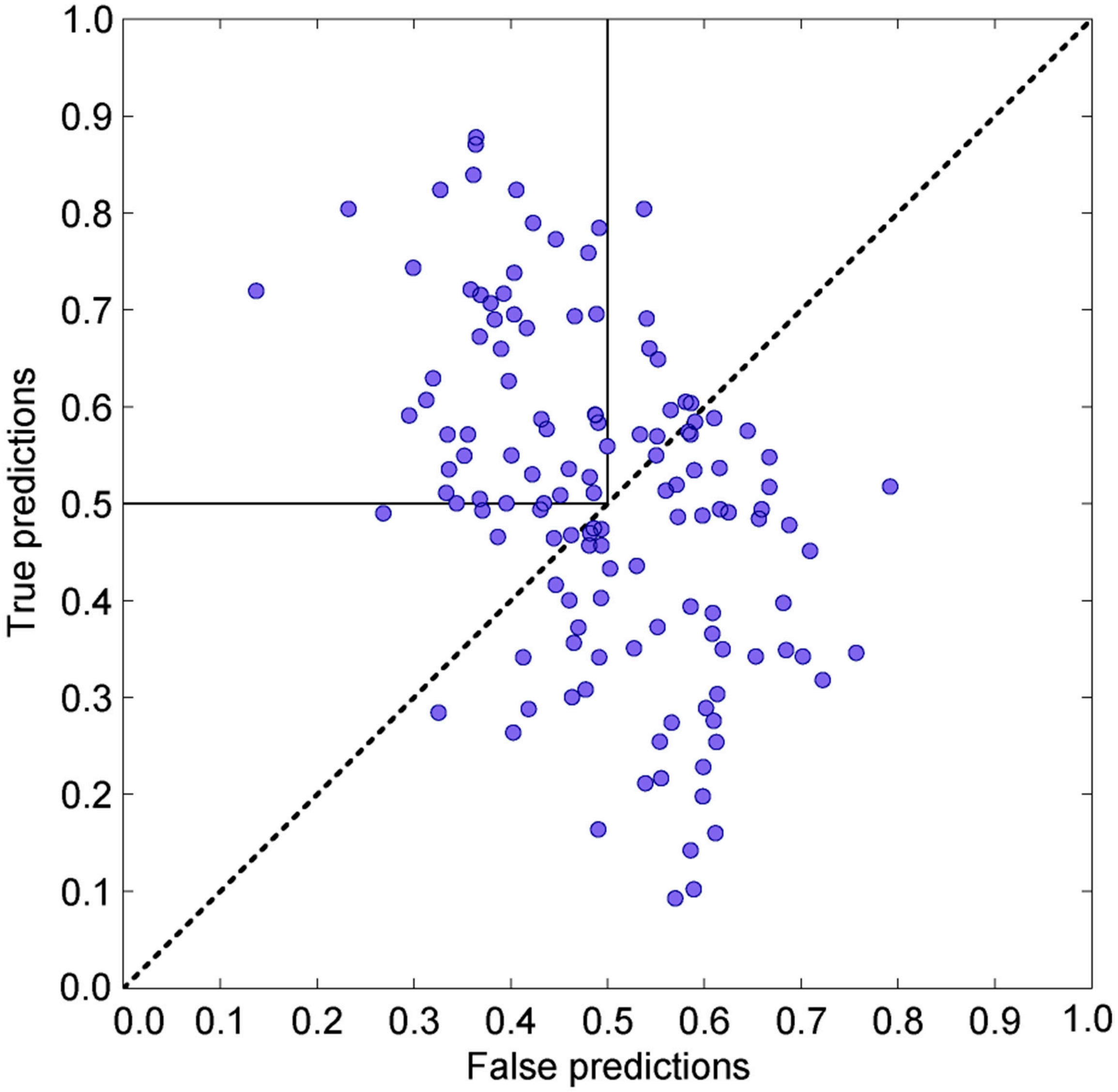

Figure S15. Prediction output based on the approach that uses an adjustable number of fastest modes per chain and sequential influence of hot residues, for low sequence-length ratio dimer chains (length ratio less than two, chain length greater than 80 residues). The true positives mean true is 50.39 %, and the false positives mean is 49.53 %. There is 34.85 % of good predictions (46 chains) and 27.27 % of very bad predictions (36 chains).

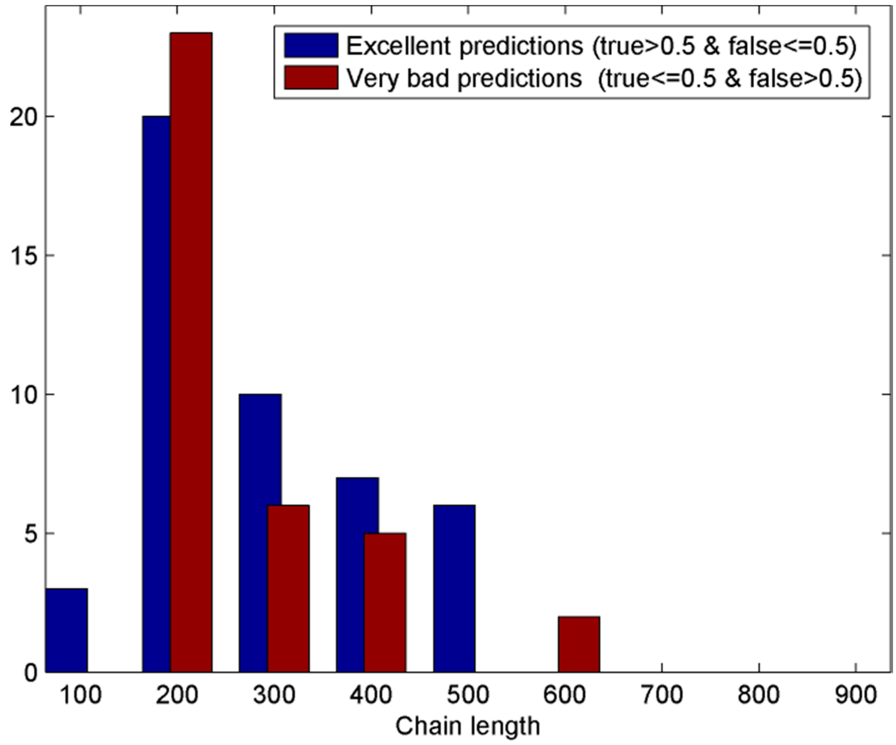

Figure S16. Prediction histogram over the sequence lengths for the prediction approach based on the adjustable number of fast modes, for the 1D influence of hot residues, for chains in dimers with low sequence length ratio (length ratio <= 2, length > 80 residues). There is 34.85 % of good predictions (46 of 132 chains) and 27.27 % of very bad predictions (36 chains).

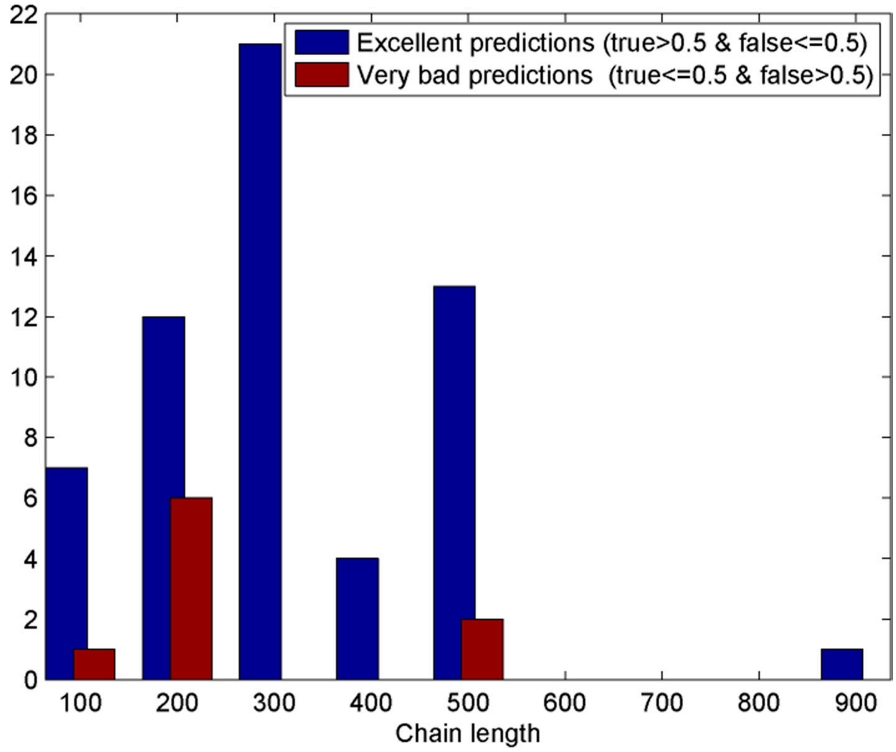

Figure S17. Prediction histogram over the sequence lengths for the prediction approach based on the adjustable number of fast modes and variable 3D influence per hot residue, for chains in dimers with high sequence length ratio (length ratio > 2, length > 80 residues). The true positives mean is 53.77 %, and the false positives mean is 41.29 %. There is 56.31 % of good predictions (58 chains) and 8.74 % of very bad predictions (9 chains).

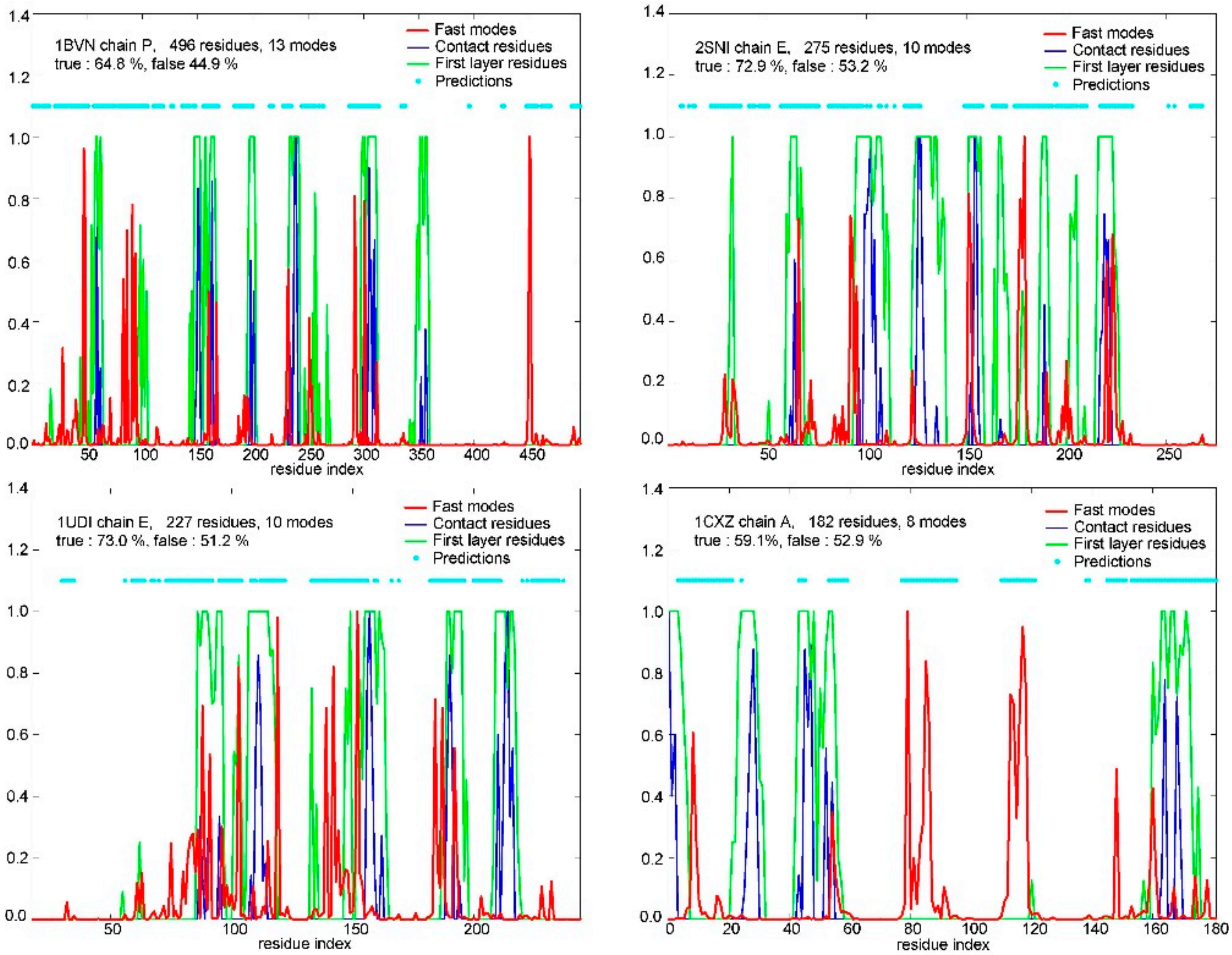

Figure S18. Examples of the prediction based on the adjustable number of fast modes and the sequential influence of hot residues. The four different chains are depicted (1BVN chain P, 2SNI chain E, 1UDI chain E and 1CXZ chain A). Red lines depict weighted sums. Blue lines designate contacts residues. Green lines are first layer residues. Cyan dots are predictions. For the three longest chains from that group, 1BVN chain P, 2SNI chain E, 1UDI chain E, the percent of true positives is over 60%, and percent of false positives is about 50 % or less (the chain E of 1UDI, has a highest difference between true and false positives which is an indication of a high correlation between the kinetically hot residues and contact scaffolds for that chain). Only the shortest example, 1CXZ chain A, has both true and false positives over 50 %.

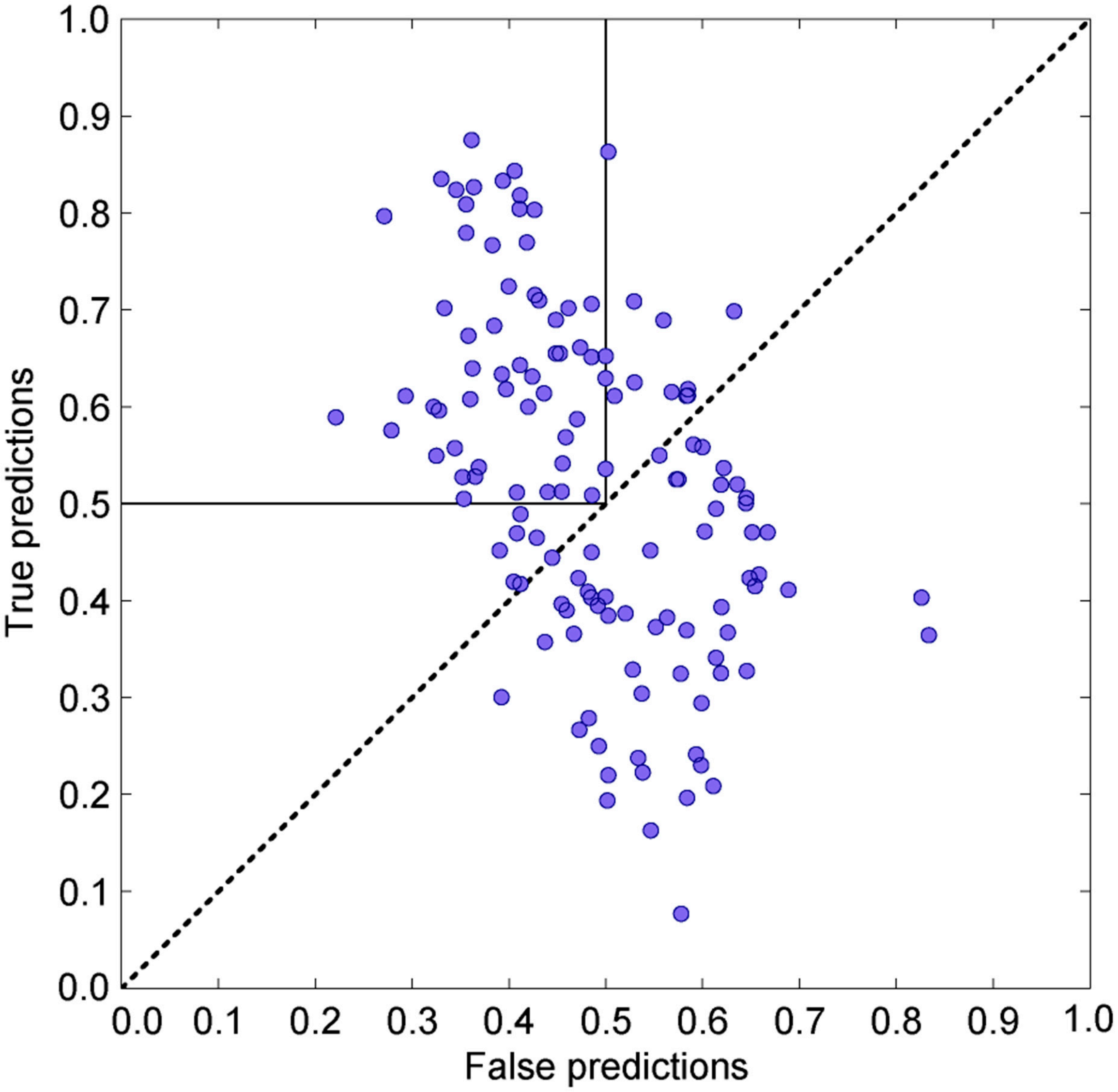

Figure S19. Prediction output for the prediction approach based on the adjustable number of fastest modes per chain and the variable 3D influence per hot residue, for chains in dimers with low sequence length ratios (length ratio less than 2, chain length > 80 residues). The true positives mean is 52.22 %, and false positives mean is 48.81 %. There is 42.42 % of good predictions (56 chains) and 27.27 % of very bad predictions (36 chains).

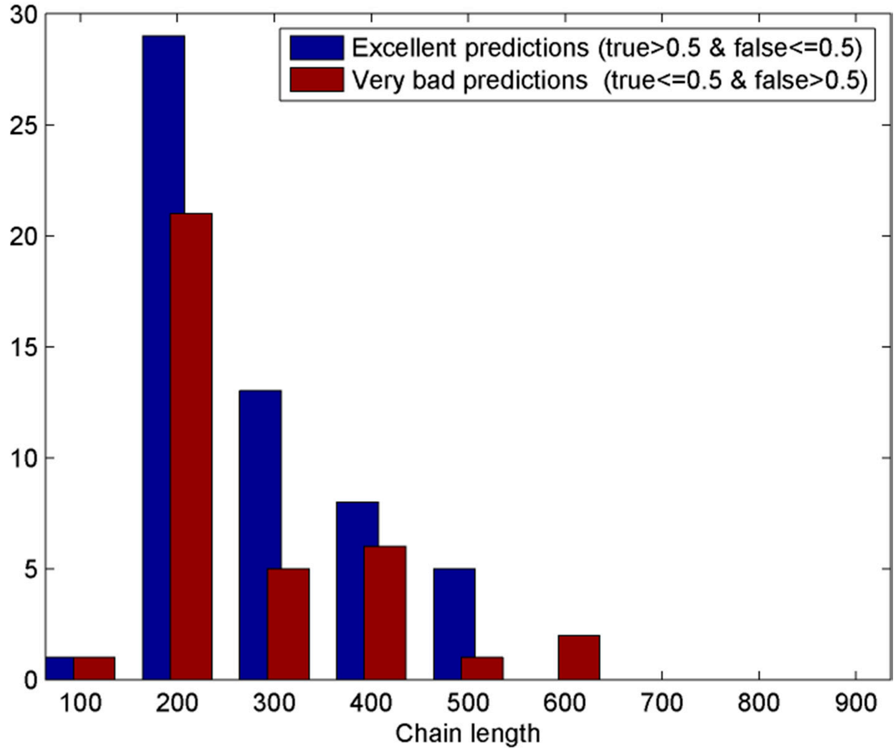

Figure S20. Prediction histogram over the sequence lengths for the prediction approach based on the adjustable number of fast modes and the variable 3D influence per hot residue, for chains in dimers with low sequence length ratio (length ratio < 2, length > 80 residues). The true positives mean is 52.22 %, and false positives mean is 48.81 %. There is 42.42 % of good predictions (56 chains) and 27.27 % of very bad predictions (36 chains).

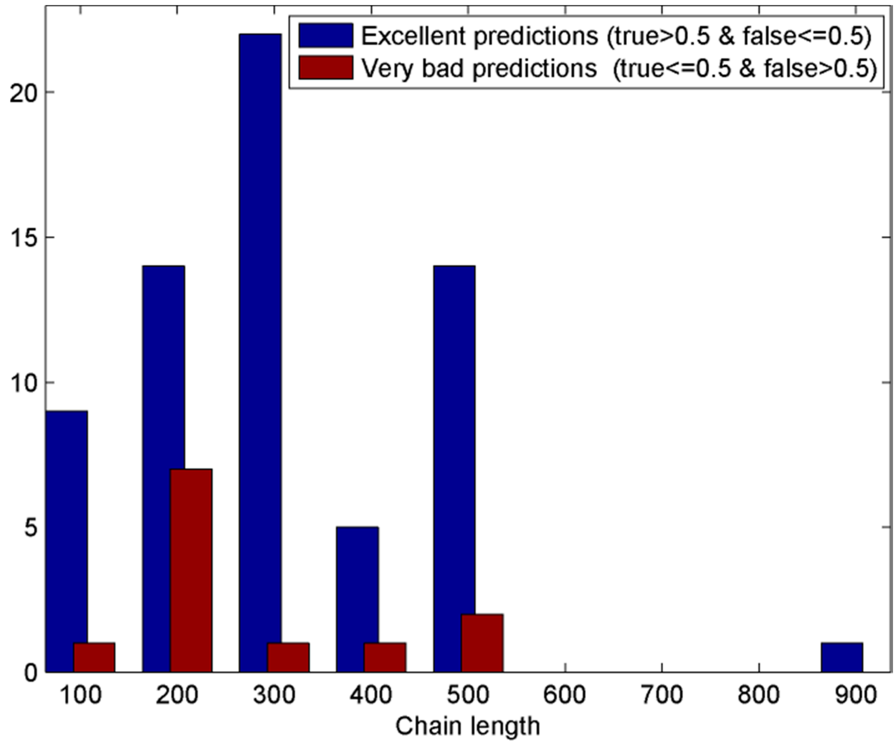

Figure S21. Prediction histogram over the sequence lengths for the prediction approach based on the adjustable number of fast modes and combined 1D & fixed 3D influence per hot residue for chains in dimers with high sequence length ratio (length ratio higher than 2, length > 80 residues). The influence is first spread linearly, upstream and downstream along the sequence, and then the it is spread to residue's spatial neighbors, the ones closer than 6 or 8 Å). True positives mean is 56.77 %, and the false positives mean is 43.21 %. There is 63.11 % of good predictions (65 chains) and 11.65 % of very bad predictions (12 chains).

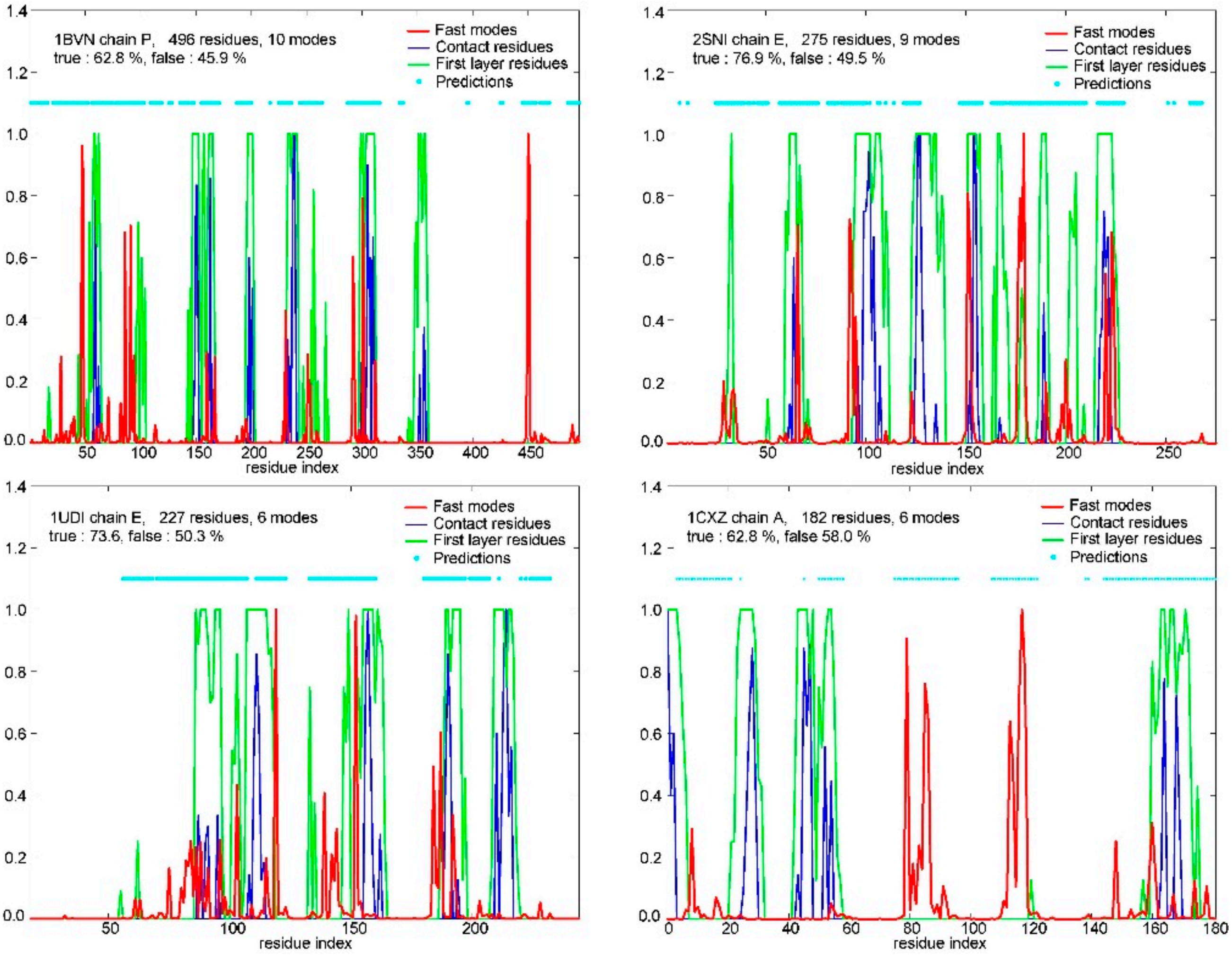

Figure S22. Examples of the prediction based on the adjustable number of fast modes and combined 1D & 3D influence per hot residue. The four different chains are depicted (1BVN chain P, 2SNI chain E, 1UDI chain E and 1CXZ chain A). Red lines depict weighted sums. Blue lines designate contacts residues. Green lines are first layer residues. Cyan dots are predictions. For the three longest chains from that group, 1BVN chain P, 2SNI chain E, 1UDI chain E, the percent of true positives is over 60%, and percent of false positives is about 50 % or less (the chain E of 2SNI, has a highest difference between true and false positives which is an indication of a high correlation between the kinetically hot residues and contact scaffolds for that chain). Only the shortest example, 1CXZ chain A, has both true and false positives over 50 %.

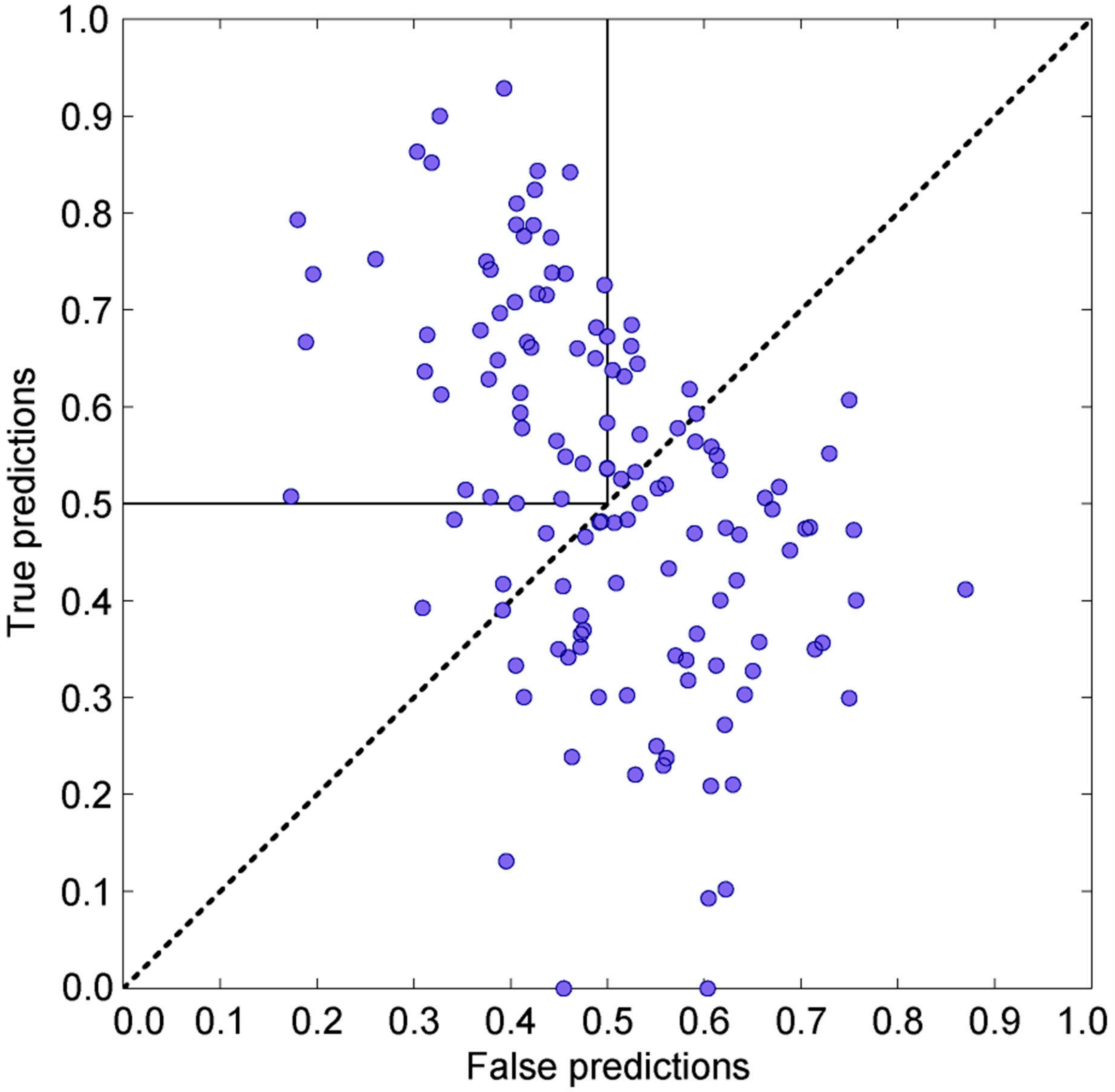

Figure S23. Prediction output for the prediction approach based on the adjustable number of fastest modes per chain and combined 1D & 3D influences of hot residues, for chains in dimers with low sequence length ratio (length ratio < 2, length > 80 residues). The true positives mean is 51.57 %, and the false positives mean is 50.00 %. There is 37.88 % of good predictions (50 chains) and 29.55 % of very bad predictions (39 chains).

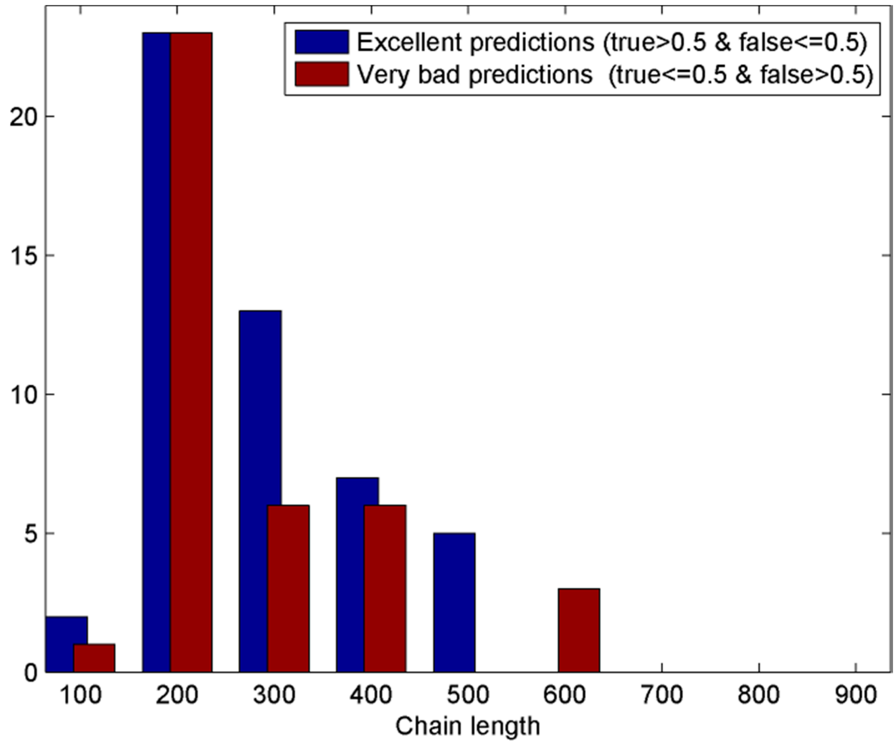

Figure S24. Prediction histogram over the sequence lengths for the prediction approach based on the adjustable number of fast modes and combined 1D & fixed 3D influence per hot residue for chains in dimers with low sequence length ratio (length ratio < 2, length > 80 residues). The influence is first spread linearly, upstream and downstream along the sequence, and then the it is spread to residue's spatial neighbors, the ones closer than 6 or 8 Å). The true positives mean is 51.57 %, and the false positives mean is 50.00 %. There is 37.88 % of good predictions (50 chains) and 29.55 % of very bad predictions (39 chains).

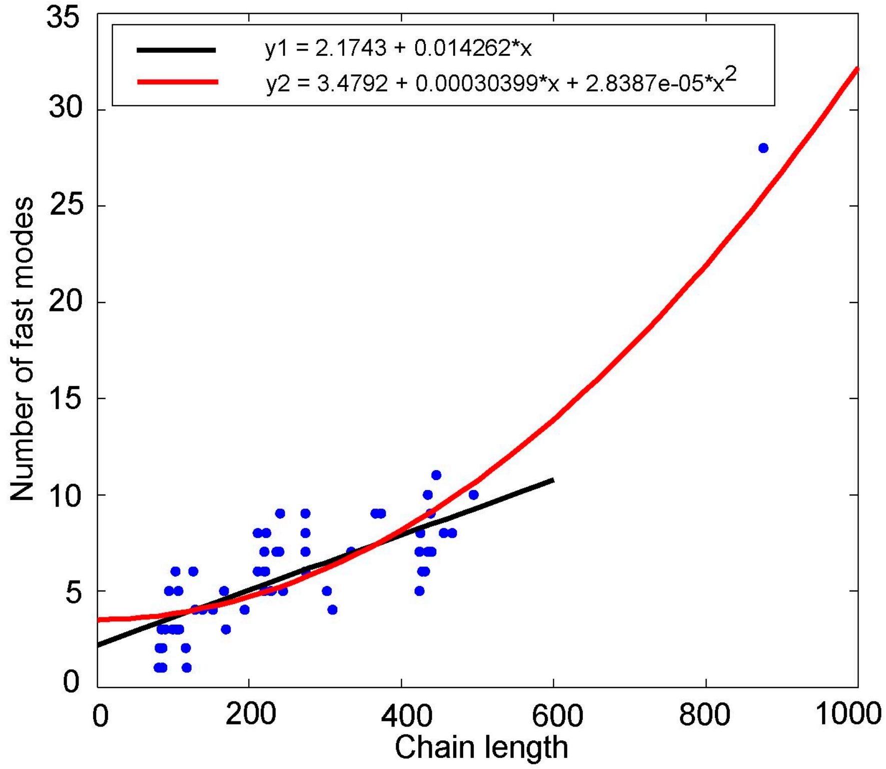

Figure S25. Linear and quadratic relationships of the number of modes per chain, for successfully characterized heterodimer chains from dimers with high sequence length ratios.

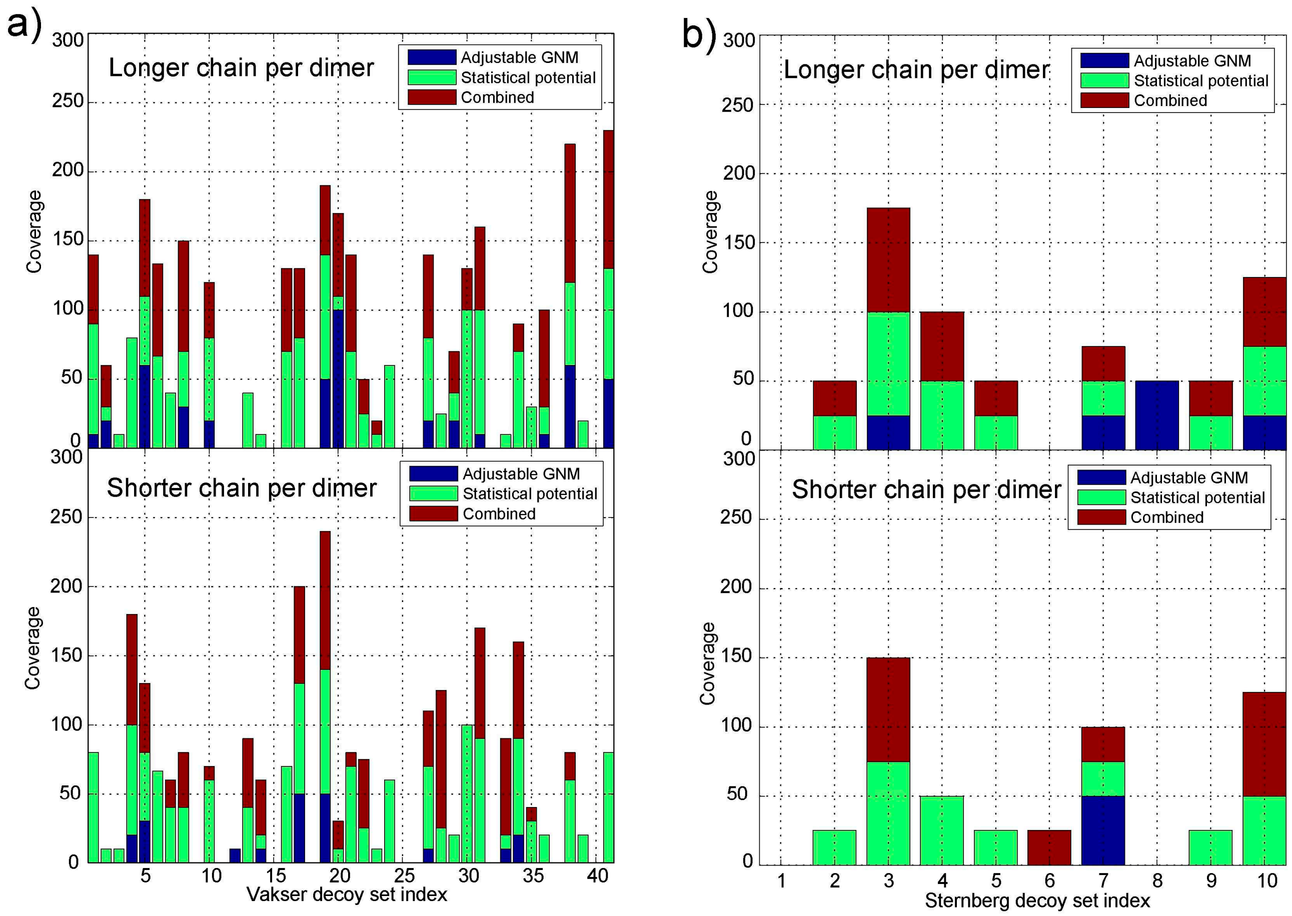

Figure S26. Comparison of the abilities of the adjustable 3D GNM approach, the statistical potential and their combination to distinguish near native decoys from the false decoys. The ability is expressed as the percent of correctly predicted near native structures among the first *n* structures, where *n* is the number of near native structures. The taller the bar, the better is the prediction. The upper plot correspond to longer chains, and the lower plot to their shorter partners. The plots on the left correspond to Vakser decoy sets and the plots on the right to Sternberg decoy sets.

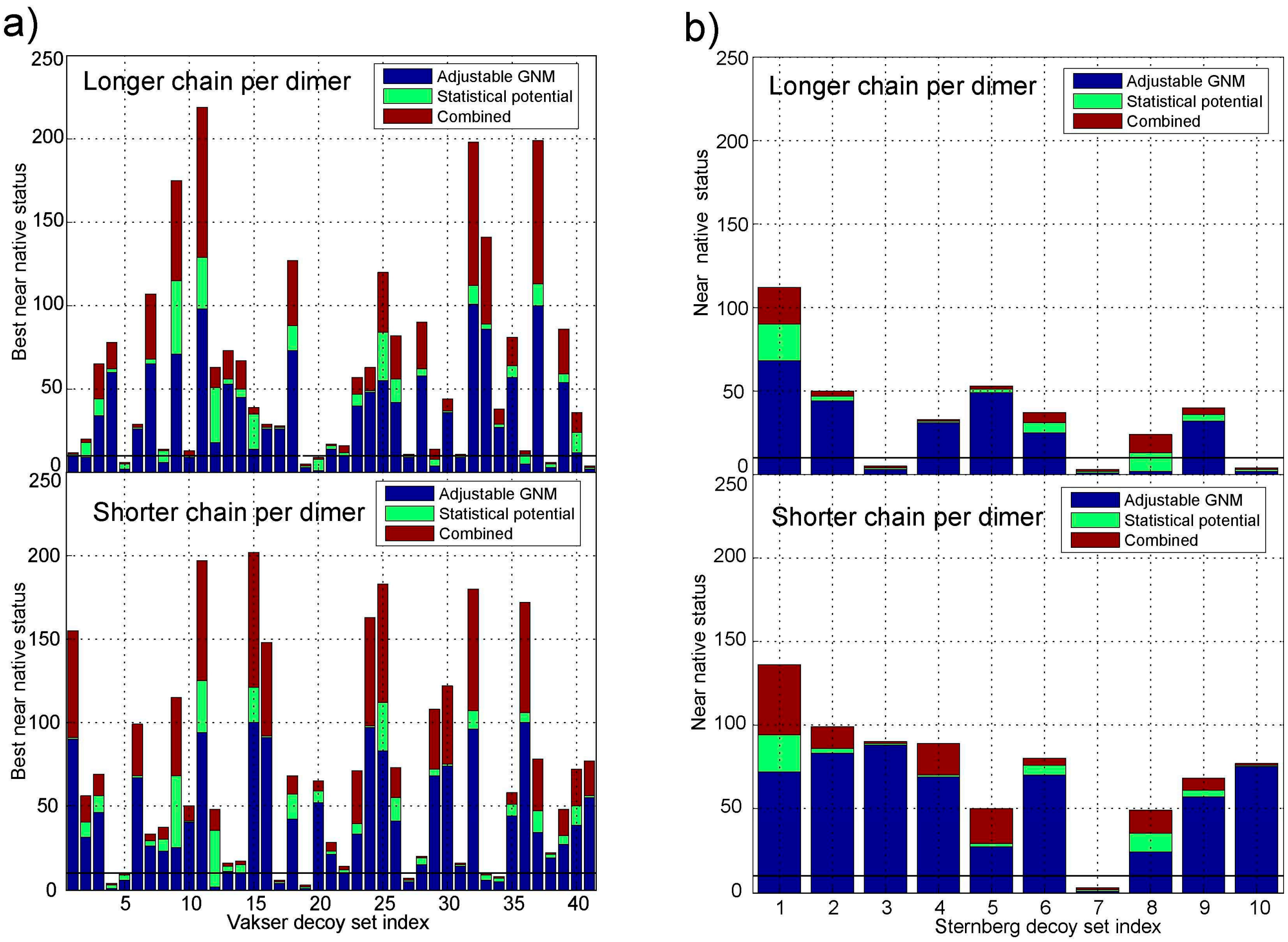

Figure S27. Comparison of the abilities of the adjustable 3D GNM approach, the statistical potential and their combination to distinguish near native decoys from the false decoys. The status of the best near native structure for each decoys set is depicted as a vertical bar. The shorter the bar, the better the prediction.
